# Morin as a Modulator of Hepatic Glucose Fluxes: A Balance Between Antihyperglycemic Potential and Mitochondrial Toxicity

**DOI:** 10.1002/jbt.70386

**Published:** 2025-06-30

**Authors:** Letícia Fernanda Nanami, Eduardo Makiyama Klosowski, Márcio Shigueaki Mito, Giovana Natiele Machado Esquissato, Gabriel Arcanjo Viana, Ana Clara Oliveira Abido, Mariane Carneiro da Silva, Ana Paula da Silva Mendonça, Gabriele Sauthier Romano de Melo, Paulo Sérgio Alves Bueno, Francielle Pelegrin Garcia, Danielle Lazarin Bidoia, Tânia Ueda Nakamura, Celso Vataru Nakamura, Emy Luiza Ishii‐Iwamoto, Ana Paula Ferro, Wanderley Dantas dos Santos, Osvaldo Ferrarese‐Filho, Rogério Marchiosi, Rodrigo Polimeni Constantin

**Affiliations:** ^1^ Laboratory of Biological Oxidations, Department of Biochemistry State University of Maringá Maringá Paraná Brazil; ^2^ Laboratory of Plant Biochemistry, Department of Biochemistry State University of Maringá Maringá Paraná Brazil; ^3^ Laboratory of Molecular Biology of Prokaryotes, Department of Biochemistry State University of Maringá Maringá Paraná Brazil; ^4^ Laboratory of Technological Innovation in the Development of Pharmaceuticals and Cosmetics, Department of Basic Health Sciences State University of Maringá Maringá Paraná Brazil

**Keywords:** antihyperglycemic agents, flavonoids, liver metabolism, mitochondrial toxicity, toxicological potential

## Abstract

This study evaluated the acute effects of morin on gluconeogenesis and glycogenolysis, key metabolic pathways that maintain glycemia, in perfused rat livers. It also assessed the acute effects of morin on mitochondrial energy metabolism and toxicity in hepatic cancer cells (HepG2) and renal epithelial cells (VERO), alongside its impact on the activity of key enzymes. Liver perfusion experiments assessed glucose fluxes, oxygen consumption, adenine nucleotide levels, and enzyme activities. Isolated mitochondria evaluated the effects of morin on oxidative phosphorylation. Enzymatic assays and MTT tests conducted in vitro determined the effects on hepatic enzymes and cell viability. In perfused rat livers, morin generally inhibited gluconeogenesis from various substrates, stimulated glycogenolysis and glycolysis, and altered oxygen consumption. Experiments on morin biotransformation suggested that this process may contribute to the inhibition of gluconeogenesis. Moreover, morin inhibited citric acid cycle activity under gluconeogenic conditions and reduced cellular ATP/ADP and ATP/AMP ratios under both gluconeogenic and glycogenolytic conditions. The elevated activity of cytosolic and mitochondrial enzymes in the effluent from perfused livers indicated impaired membrane integrity. In isolated rat liver mitochondria, morin inhibited the electron transport chain, the ATP/ADP exchange system, and functioned as an uncoupling agent of oxidative phosphorylation, thereby reducing ATP synthesis. Under in vitro conditions, morin inhibited the activity of glucose 6‐phosphatase, glucokinase, glucose 6‐phosphate dehydrogenase, and pyruvate kinase from rat livers. At the cellular level, morin decreased the viability of HepG2 and VERO cells, indicating its toxicity. The increased glucose release due to heightened glycogenolysis, combined with the suppression of gluconeogenesis, may impact the expected antihyperglycemic effects of morin. These outcomes were partly attributed to mitochondrial bioenergetic disruption, which is an important consideration for the therapeutic use of morin, particularly with prolonged treatment or higher doses. Together, these findings highlight morin's potential as an antihyperglycemic agent but also reveal significant concerns regarding its mitochondrial toxicity.

## Graphical abstract

Morin modulates hepatic glucose metabolism by inhibiting gluconeogenesis and enhancing glycogenolysis in perfused rat livers. These effects, partly attributed to mitochondrial dysfunction and impaired oxidative phosphorylation, are accompanied by decreased ATP levels and enzyme inhibition, raising concerns about bioenergetic imbalance and potential toxicity in therapeutic contexts involving higher doses or prolonged exposure.

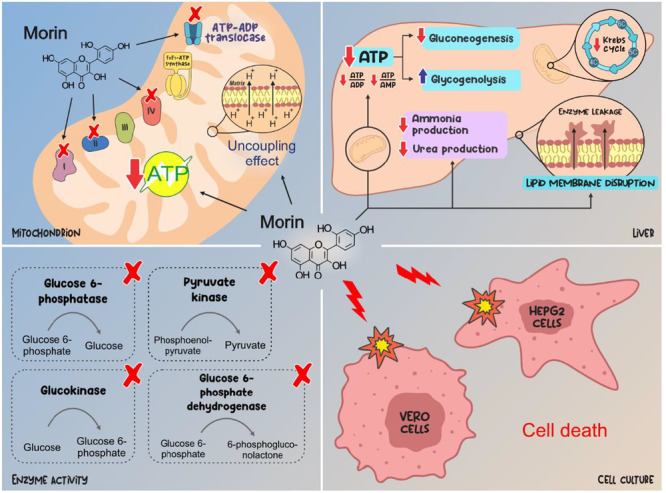

**Abbreviations**
ADP: adenosine diphosphate
ALP: alkaline phosphatase
ALT: alanine aminotransferase
AMP: adenosine monophosphate
AST: aspartate aminotransferase
ATP: adenosine triphosphate
CO_2_
: carbon dioxide
DMSO: dimethyl sulfoxide
FUM: fumarase
HCT: human colon tumor (cell line)
HepG2: hepatic cancer cells
HPLC: high‐performance liquid chromatography
IC_50_
: half maximal inhibitory concentration
K_M_
: Michaelis–Menten constant
LDH: lactate dehydrogenase
MPT: mitochondrial permeability transition
MTT: 3‐(4,5‐dimethylthiazol‐2‐yl)−2,5‐diphenyltetrazolium bromide
NADH: nicotinamide adenine dinucleotide (reduced form)
NADPH: nicotinamide adenine dinucleotide phosphate (reduced form)
OV: ovarian (related to cancer cell lines)
PTP: permeability transition pore
RC: respiratory control ratio
SK: SK‐OV‐3 ovarian cancer cell line
TMPD: N,N,N′,N′‐tetramethyl‐p‐phenylenediamine
TOV: TOV‐21G ovarian cancer cell line
UDP: uridine diphosphate
VERO: kidney epithelial cells from African green monkey

## 1 Introduction

Natural products have emerged as a central focus of scientific research, driving significant advancements in modern society. These molecules are intricately linked to the presence of various substituents (chemical groups), enabling specific interactions with macromolecules, involvement in signaling cascades, and modulation of membrane permeability and fluidity [1]. Extracts, phenolic compounds, flavonoids, carotenoids, anthocyanins, and saponins are now regarded as valuable compounds for disease treatment. As a result, nutraceuticals and herbal remedies have increased, driven by their lower toxicity, accessibility, and tendency to induce milder side effects compared to synthetic medications [2]. In the context of escalating exploration of natural compounds and secondary metabolites, there is a notable surge in research directed towards flavonoids. These polyphenolic compounds, abundant in plants, fruits, and roots, have garnered attention for their exclusive biosynthesis by plants. Despite originating from plants, flavonoids exhibit a wide range of biological and pharmacological effects on animal cells [3]. Flavonoids are increasingly recognized as promising dietary supplements due to their potential benefits in glucose homeostasis. Research suggests that these polyphenols can inhibit carbohydrate‐metabolizing enzymes, modulate glucose absorption, and regulate insulin secretion via diverse signaling pathways. Such properties hold notable promise as a therapeutic strategy for diabetes management [4, 5].

**Figure 1:**
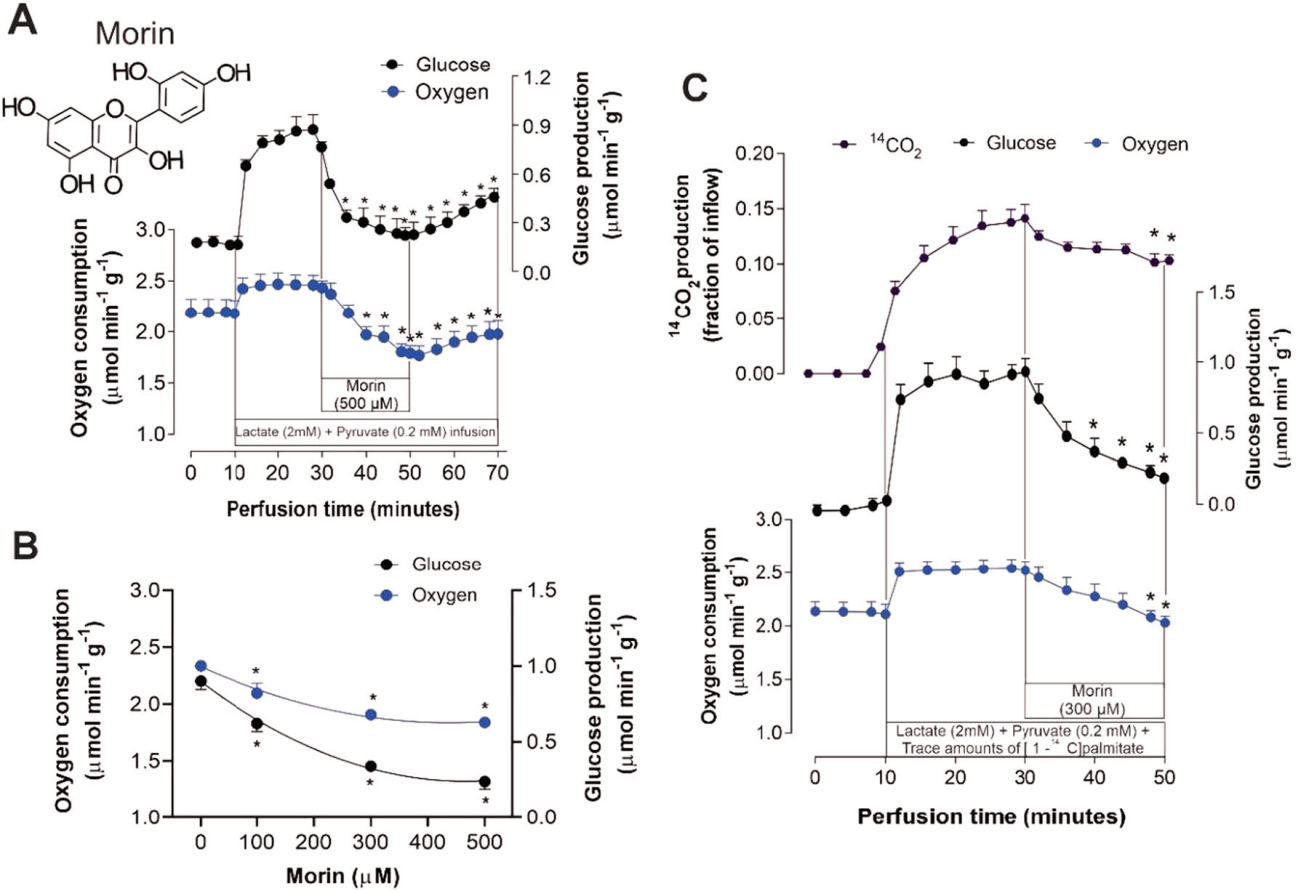
Chemical structure of morin and its effects on the metabolic fluxes of lactate and pyruvate in isolated perfused livers from fasting rats. (A) Temporal evolution of changes induced by 500 μM morin in glucose production and oxygen consumption. (B) Concentration‐dependent effects of morin on glucose production and oxygen consumption. These effects were determined as the difference between the rates in the presence of lactate + pyruvate alone and the minimum rates following the onset of morin infusion. (C) Time courses of changes induced by 300 μM morin in glucose production, oxygen consumption, and ^14^CO_2_ production from traces of [1‐^14^C]palmitate. Each experimental point represents the mean of four independent experiments, and the bars indicate the standard errors of the mean. *p < 0.05, ANOVA with Dunnett's post hoc test.

Morin (3,5,7,2ʹ,4ʹ‐pentahydroxyflavone; see inset in Figure 1A) is a flavonoid classified under the flavonol group commonly found in plants of the *Moraceae*, such as fig trees. It is also present in various fruits and vegetables, including almond skins, guava leaves (*Psidium guajava*), black mulberries (*Maclura tinctoria*), osage oranges (*Maclura pomifera*), and red onions (*Allium cepa*). Due to its widespread occurrence, morin plays a significant role in botanical health practices [3, 6, 7, 8]. This flavonol is distinguished by the presence of a hydroxyl group at position 3, which forms part of the flavone structure (2‐phenyl‐1‐benzopyran‐4‐one), as well as being a 7‐hydroxyflavonol with three additional hydroxyls at positions 2ʹ, 4ʹ, and 5. In its pure state, morin has a bitter taste and a yellowish hue, making it a traditional dye for cotton, wool, and silk. Morin has limited solubility in water at room temperature but dissolves well in ethanol, alcohol, and organic solvents [2].

Prior research has highlighted morin's diverse biological activities in both in vitro and in vivo settings, including its antioxidant properties, anti‐inflammatory effects, hepatoprotective activity, and anticarcinogenic potential [9, 10, 11, 12, 13, 14, 15, 16]. Furthermore, morin offers numerous health benefits, potentially serving as an antihyperglycemic agent while aiding in the prevention of obesity and diabetes [17, 18, 19, 20]. For instance, a previous study reported that oral administration of a zinc‐morin complex to diabetic rats (5 mg/kg body weight/day for 30 days) led to reduced blood glucose and glycated hemoglobin (HbA1c) levels. Additionally, the study found that morin treatment effectively improved glucose intolerance and insulin resistance [21]. Another study on diabetic rats revealed that treatment with morin (at doses of 25 and 50 mg/kg for 30 days) ameliorated metabolic dysregulation induced by diabetes. This included reductions in blood glucose levels, enhancement of serum insulin levels via β‐cells, and modulation of carbohydrate metabolism, all of which affected the activities of glucose 6‐phosphatase and fructose 1,6‐bisphosphatase [17]. Research also underscores morin's role in enhancing glycogen synthesis, inhibiting gluconeogenesis, and increasing the phosphorylation levels of Akt and insulin receptors in HepG2 cell cultures [22]. Moreover, morin treatment downregulates the expression level of miR‐29a, thereby improving insulin signaling and glucose metabolism in HepG2 cell cultures [23]. Morin enhances insulin receptor phosphorylation, promoting insulin sensitivity and glycogen synthesis while inhibiting gluconeogenesis in high‐fat diet‐induced obese mice [24]. More recently, a study showed that morin‐based nanoparticles encapsulated in sodium alginate microgels significantly enhanced glucose uptake in HepG2 cells. These nanoparticles also effectively managed blood glucose levels, lipid profiles, and oxidative stress in diabetic mice, while concurrently mitigating liver, kidney, and pancreatic damage and promoting anti‐inflammatory responses [25].

Collectively, the aforementioned findings unequivocally underscore the beneficial role of morin in managing diabetes mellitus and related disorders. However, despite significant progress made in recent years in elucidating the mechanisms underlying morin's antihyperglycemic effects, these mechanisms remain numerous and are not yet fully understood. To our knowledge, no existing study has documented the acute effects of morin on hepatic metabolic pathways involved in glucose homeostasis, such as gluconeogenesis and glycogenolysis. Current findings are primarily based on chronic morin treatment and, in some cases, do not involve the use of an intact liver, limiting the generalizability of the results to physiological conditions. Compelling evidence suggests that both gluconeogenesis and glycogenolysis can be inappropriately elevated in individuals with type 2 diabetes, contributing to persistent hyperglycemia and increasing the risk of diabetes‐related complications [26, 27, 28, 29, 30]. Therapeutic agents designed for diabetes management can regulate glycemic levels by acutely inhibiting hepatic glycogenolysis and/or gluconeogenesis [29]. Given this, it is crucial to explore the potential acute effects of morin on these metabolic pathways. Therefore, the primary aim of the present study was to investigate the acute effects of morin using the isolated perfused rat liver as the experimental system. This system allows for the simultaneous monitoring of multiple interconnected metabolic pathways while preserving the integrity of microcirculation and intercellular relationships, thus providing a closer approximation of physiological conditions [26, 31, 32].

Despite its numerous benefits for metabolism and energy homeostasis, morin has also been linked to some toxic effects. For instance, a 13‐week dietary toxicity study of morin (0%–5%, w/w) in F344 rats revealed significant elevations in alanine transaminase (ALT), aspartate transaminase (AST), alkaline phosphatase (ALP), γ‐glutamyl transpeptidase (γ‐GT), and relative liver weights [33]. Another study demonstrated morin's ability to reduce cell viability and induce caspase‐dependent apoptosis in HCT‐116 cells [34]. Bieg and colleagues examined the synergistic effects of morin and cisplatin on TOV‐21G (cisplatin‐sensitive) and SK‐OV‐3 (cisplatin‐resistant) ovarian cancer cells. According to their results, morin effectively exerted anticancer effects against SK‐OV‐3 and TOV‐21G cells by increasing apoptosis and decreasing cell viability and proliferation [35]. In another study, morin prominently reduced the Bax/Bcl‐2 ratio induced by 1,2‐dimethylhydrazine, highlighting its proapoptotic activity [36]. Besides these possibilities, a particular toxicological effect associated with morin could theoretically result in impaired mitochondrial energy transduction. In an early study involving beef heart mitochondria, Lang and Racker showed that morin inhibited the ATPase activity of the F_o_F_1_‐ATP synthase complex at concentrations as low as 0.1 μM [37]. Supporting this possibility, fisetin and quercetin—flavonoids structurally similar to morin—have been found to disrupt mitochondrial energy transduction, impairing ATP synthesis via oxidative phosphorylation. This deleterious effect on mitochondrial energy transduction can be regarded, at least partially, as a causative factor for the metabolic alterations induced by these flavonoids in the liver [38, 39, 40]. Given the structural similarity between morin and these active flavonoids, we hypothesized that morin may exert similar effects on mitochondrial bioenergetics, potentially influencing metabolic fluxes related to glucose metabolism in the liver. Therefore, a comprehensive investigation into morin's acute toxic effects on bioenergetics‐related parameters was conducted in isolated mitochondria to furnish supporting evidence and enhance the understanding of the overall findings of the present study. Because morin has been shown to reduce cell viability [34, 35, 36], we expanded our study to assess its acute impact on the viability of hepatic cancer cells (HepG2) and renal epithelial cells (VERO), further complementing previous research. We anticipate these outcomes to enrich our comprehension of morin's mechanism action against hyperglycemia, and guide decisions about its application in treating type 2 diabetes mellitus, with careful attention to its safety implications.

## 2 Materials and Methods

### 2.1 Chemicals

Morin (MF: C_15_H_10_O_7_; MW: 302.23 g/mol; PubChem CID 5281670) was obtained from Sigma‐Aldrich (St. Louis, MO, USA). The [1‐^14^C]palmitic acid, with a specific activity of 60.0 mCi/mmol, was obtained from PerkinElmer. Substrates, enzymes, and coenzymes were sourced from Sigma‐Aldrich. Fetal bovine serum (FBS), penicillin, and 3‐[4,5‐dimethylthiazol‐2‐yl]−2,5‐diphenyl tetrazolium bromide (MTT) were sourced from Invitrogen. All other chemicals used were of the highest commercial grade available.

### 2.2 Animals

Male albino Wistar rats, aged 49 days and weighing between 180 and 220 g, were randomly selected from the Central Vivarium of the State University of Maringá. They were kept in polycarbonate cages at the Sectorial Vivarium of the Department of Biochemistry. Environmental conditions were controlled, maintaining a temperature of 20°C–23°C and a 12‐h light/dark cycle (lights on at 6:00 a.m.) to mimic natural day and night patterns. The rats had unrestricted access to standard laboratory chow (Nuvilab CR‐1, Colombo, Brazil) and water. A 7‐day acclimation period preceded the start of the experiments. For experiments assessing gluconeogenesis, glycolysis from exogenous glucose, and evaluating outflow profiles and morin biotransformation, the rats were fasted for 12 h (overnight) before analysis. On the day of the experiments, the rats were anesthetized via intraperitoneal injection consisting of ketamine (80 mg/kg), xylazine (10 mg/kg), and fentanyl (0.06 mg/kg). All experimental procedures adhered to internationally recognized guidelines for the care and use of animals. Approval for the study was obtained from the Ethics Committee for Animal Experiments at the State University of Maringá (CEUA‐UEM, protocol number 2483170517).

### 2.3 Liver Perfusion and Metabolite Measurement

A hemoglobin‐free, nonrecirculating liver perfusion was carried out following a standardized protocol [41, 42]. Based on experimental requirements, specific substrates such as lactate, pyruvate, fructose, glucose, alanine, dihydroxyacetone, and glycerol were added directly to the perfusion fluid and dissolved without using any adjuvants. When required, the mitochondrial respiratory chain in perfused livers was inhibited by the infusion of 2 mM sodium cyanide, which was also dissolved in the perfusion fluid without adjuvants. Morin was dissolved in the perfusion fluid by simultaneously adding an equivalent amount of 1.0 M NaOH, and the pH was then adjusted to 7.6. Effluent perfusion fluid samples were collected at intervals of 2–4 min and subsequently analyzed for metabolite concentrations. Lactate, pyruvate, ammonia, and urea concentrations were assessed through established enzymatic methods [42, 43]. Potential interference from morin (absorbance at 340 nm) was mitigated by running appropriate blank samples. Glucose concentrations were determined colorimetrically using the *o*‐toluidine method [44]. Polarographic monitoring was utilized for assessing the oxygen concentration in the outflowing perfusate [42, 45]. To quantify ^14^CO_2_ produced from [1‐^14^C]palmitate, ^14^CO_2_ was trapped using phenylethylamine, and radioactivity was measured via liquid scintillation spectroscopy [40, 46, 47]. Hepatic adenine nucleotides (AMP, ADP, and ATP) were quantified by HPLC after freeze‐clamping the perfused livers with liquid nitrogen [48]. The concentrations of morin used in this study (100–500 μM) were selected based on previously published investigations from our research group and others, which have utilized flavonoids and related xenobiotics at similar concentrations in isolated perfused rat liver experiments [26, 38, 40, 49, 50, 51]. Moreover, in liver perfusion models, the need to saturate hepatic uptake and simulate pharmacological exposure justifies higher experimental concentrations.

### 2.4 Evaluation of Outflow Profiles for Morin

The evaluations were conducted under two distinct conditions: (a) livers from fasted rats perfused with morin at a concentration of 100 μM, and (b) livers from fed rats perfused with morin at the same concentration. Following a pre‐perfusion period of 10 min, morin was infused for 40 min, followed by an additional 10 min of perfusion without morin. The perfusate effluent was fractioned by HPLC. Samples were filtered through a 0.45 μM disposable syringe filter before analysis (20 μL) with an HPLC system. The analyses were performed using a Shimpack CLC‐ODS (M) reversed‐phase column (250 × 4.6 mm, 5 μM particle size), paired with an equivalent precolumn (10 × 4.6 mm). The mobile phase consisted of a methanol/water/acetic acid mixture (40:58:2, v/v/v). The HPLC system was operated isocratically at a flow rate of 1.0 mL/min at 55°C, with spectrophotometric monitoring at 288 nm. Morin concentrations in each perfusate sample were calculated using regression parameters derived from a calibration curve. A linear relationship between concentration and the areas under the curves was observed. Morin levels detected in the perfusate were expressed as a fraction of the venous/portal concentration [52].

### 2.5 Mitochondria Isolation and Preparation for the Evaluation of Respiratory Function and Enzyme Activity

Mitochondria were isolated from freshly excised livers obtained immediately after animal euthanasia to ensure optimal organelle integrity and functionality. The mitochondria were meticulously isolated using a differential centrifugation method, preserved at a temperature range of 0°C–4°C and, as required, subsequently disrupted by liquid nitrogen through repeated cycles of freezing and thawing [53, 54].

### 2.6 Assessment of Mitochondrial Oxygen Consumption, Respiratory Control Ratio (RC), and ADP/O Ratio

The determination of oxygen consumption by intact, coupled mitochondria was conducted using polarographic techniques [45, 54]. Morin, ranging from 0 to 500 μM, was added to the incubation medium as a solution in dimethyl sulfoxide (DMSO). Mitochondrial oxygen consumption was quantified in nmol O₂/min × mg of protein. The RC and ADP/O ratios were determined using the Chance and Williams method [55].

### 2.7 Assessment of Enzymatic Activities Bound to the Inner Mitochondrial Membrane

NADH and succinate oxidase activities, along with the activity of complex IV (cytochrome *c* oxidase), were quantified using polarographic measurement techniques. For the assessment of NADH and succinate oxidase activities and oxidation of TMPD‐ascorbate, mitochondria were previously disrupted by freeze‐thaw cycles to allow access of exogenous substrates to respiratory chain components [54, 56]. The results were expressed as nmol O₂/min × mg of protein. The ATPase activity in both intact (coupled and uncoupled) and freeze‐thaw disrupted mitochondria was measured by quantifying the release of inorganic phosphate, following a modified version of the original Pullman method [54, 57]. The amount of inorganic phosphate released into the medium was measured following the technique described by Fiske and Subbarow [58]. Data were expressed as nmol of inorganic phosphate released/min × mg of protein. In all trials, morin (0–500 μM) was consistently added as a solution in dimethyl sulfoxide (DMSO) to the incubation medium.

### 2.8 Mitochondrial Swelling

A spectrophotometric technique was utilized to observe the swelling induced by the oxidation of organic substrates, with measurements taken at a wavelength of 520 nm [59]. The reactions were commenced by the concurrent addition of 50 mM sodium acetate along with these substrates: (a) 10 mM succinate, (b) 10 mM glutamate, (c) 0.2 mM TMPD with 5 mM ascorbate, and (d) 10 mM malate. The peak absorbance amplitude and the rate of absorbance change, whether reduction or enhancement, were plotted against morin concentrations (0–500 μM) as fractions relative to the corresponding controls. In all experiments, morin (0–500 μM) was consistently introduced into the incubation medium as a solution dissolved in dimethyl sulfoxide (DMSO).

### 2.9 Assessment of Mitochondrial ATP Production

Mitochondrial ATP synthesis was determined using a previously reported method [60]. The rates of mitochondrial ATP and AMP production, along with ADP consumption, were evaluated in the presence or absence of two different concentrations of morin (150 μM and 500 μM). The levels of ATP, ADP, and AMP in the samples were analyzed via HPLC [48]. The findings were presented as nmol of ATP or AMP produced/min × mg of protein, or as nmol of ADP consumed/min × mg of protein.

### 2.10 Enzymatic Activity Assessment

For the assessment of enzyme release (LDH and fumarase), livers from fed rats were used. The objective of these experiments was to evaluate the potential of morin to induce membrane disruption and consequent leakage of cytosolic and mitochondrial enzymes. Since the structural integrity of cellular membranes is not expected to be significantly influenced by the nutritional state, the use of fasted animals was deemed unnecessary. The activities of lactate dehydrogenase (LDH) and fumarase (FUM) were measured in the outflowing perfusate, both in the presence and absence of 300 μM morin [61]. Standardized protocols were followed to measure the enzymatic activities [43]. The data were reported as μmol/min × mL outflowing perfusate.

The liver enzymes, including glucose 6‐phosphatase, glucose 6‐phosphate dehydrogenase, pyruvate kinase, fructokinase, and glucokinase, were assessed for morin sensitivity. Glucose 6‐phosphatase activity was analyzed utilizing a microsomal extract [62]. Phosphate release was measured spectrophotometrically at 700 nm [58], with rates expressed as μmol/min × mg of protein. Following the isolation of microsomes, the cytosolic fraction was employed to assess the activities of glucose 6‐phosphate dehydrogenase, pyruvate kinase, fructokinase, and glucokinase. Glucose 6‐phosphate dehydrogenase activity was determined by assessing NADPH production at 365 nm, using the methodology outlined by Bergmeyer [43]. The enzymatic activity was expressed as μmol of NADPH/min × mg of protein. Pyruvate kinase activity was assessed using spectrophotometric techniques following Bergmeyer's method [43]. The outcomes were quantified as μmol/min × mg of protein. The assessment of glucokinase activity followed a standardized methodology as described by Kuwajima et al. [63], with a focus on quantifying ATP consumption using HPLC [48]. The enzymatic results were reported as μmol/min × mg of protein. The assessment of fructokinase activity adhered to a standardized methodology, as previously described [64, 65], with a specific emphasis on measuring ADP production using HPLC [48]. In all instances, morin was dissolved in dimethyl sulfoxide (DMSO) before being added to the incubation medium to achieve final concentrations ranging from 0 to 500 μM.

### 2.11 Cytotoxicity of Morin in Cell Cultures

The cytotoxic effects of morin on renal epithelial cells (VERO) from *Cercopithecus aethiops* and human hepatic carcinoma cells (HepG2) were assessed using the MTT (3‐[4,5‐dimethylthiazol‐2‐yl]−2,5‐diphenyltetrazolium bromide) assay. VERO and HepG2 cells were cultured in Dulbecco's Modified Eagle Medium (DMEM), pH 7.2, supplemented with 10% fetal bovine serum (FBS) and penicillin. Cells were seeded at a density of 5.0 × 10^5^ cells/mL per well in 96‐well plates and exposed to increasing concentrations of morin (12.5–500 μM). Plates were then incubated for 24 h in a humidified atmosphere containing 5% CO_2_ at 37°C. Following treatment, wells were incubated with MTT (2 mg/mL) for 4 h at 37°C in the absence of light. Subsequently, the medium was aspirated, and formazan crystals were solubilized in DMSO. Absorbance was measured at 595 nm using a plate spectrophotometer (Power Wave XS—BioTek). Cell viability was calculated as a percentage relative to control conditions.

### 2.12 Statistical Analysis of Results

The data were represented using the mean and standard error of the mean (SEM). Student's *t*‐test (*p* < 0.05) was employed to compare two means, whereas one‐way ANOVA followed by Dunnett's post hoc test (*p* < 0.05) was used for comparisons involving three or more means. In specific cases, half‐maximal effective concentrations were determined. Statistical analyses were conducted using GraphPad Prism software (San Diego, CA).

## 3 Results

### 3.1 The Effects of Morin on Lactate and Pyruvate Gluconeogenesis, Alongside Its Impact on Oxygen Consumption, in the Livers of Fasted Rats

The initial experiments were designed to assess the effect of morin on the gluconeogenesis pathway. Figure 1 illustrates the findings. Figure 1A depicts a perfused liver response to a 500 μM morin infusion, outlining the experimental protocol employed in this study. Under these conditions, the infusion of lactate and pyruvate led to a significant increase in both glucose synthesis and oxygen consumption. However, infusing 500 μM morin over 20 min led to a gradual decline in glucose synthesis and oxygen consumption. The discontinuation of the morin infusion partially reversed the effects on oxygen consumption and glucose synthesis. Figure 1B presents the results from four similar experimental sets, as depicted in Figure 1A, examining different concentrations of morin (100, 300, and 500 μM). The graphs illustrate the concentration‐dependent effects of morin on glucose release and oxygen consumption, with maximum inhibition of gluconeogenesis reaching 73.41% at 500 μM morin. The inhibition of glucose synthesis showed a clear concentration dependence, with numerical interpolation indicating an IC50 of 162.66 μM for morin. Similarly, oxygen consumption inhibition also displayed a concentration‐dependent pattern, with an IC50 of 196.69 μM morin.

### 3.2 The Effects of Morin on Citric Acid Cycle Activity Under Gluconeogenic Conditions

The current study used the long‐chain fatty acid [1‐^14^C]palmitate to evaluate the citric acid cycle activity in gluconeogenic conditions. Three key parameters were assessed: oxygen consumption, glucose synthesis, and ^14^CO_2_ production (Figure 1C). When lactate and pyruvate were infused in the presence of [1‐^14^C]palmitate, a notable increase in oxygen consumption, glucose synthesis, and ^14^CO_2_ production was observed. However, the infusion of morin at 300 μM led to a gradual decrease in all three parameters. By the later stages of the experiment, this inhibition reached maximum values of 20% for oxygen consumption, 60.8% for glucose production, and 27% for ^14^CO_2_ generation.

### 3.3 The Effects of Morin on Gluconeogenesis From Fructose, the Production of Lactate and Pyruvate, and Oxygen Consumption in the Livers of Fasted Rats

**Figure 2:**
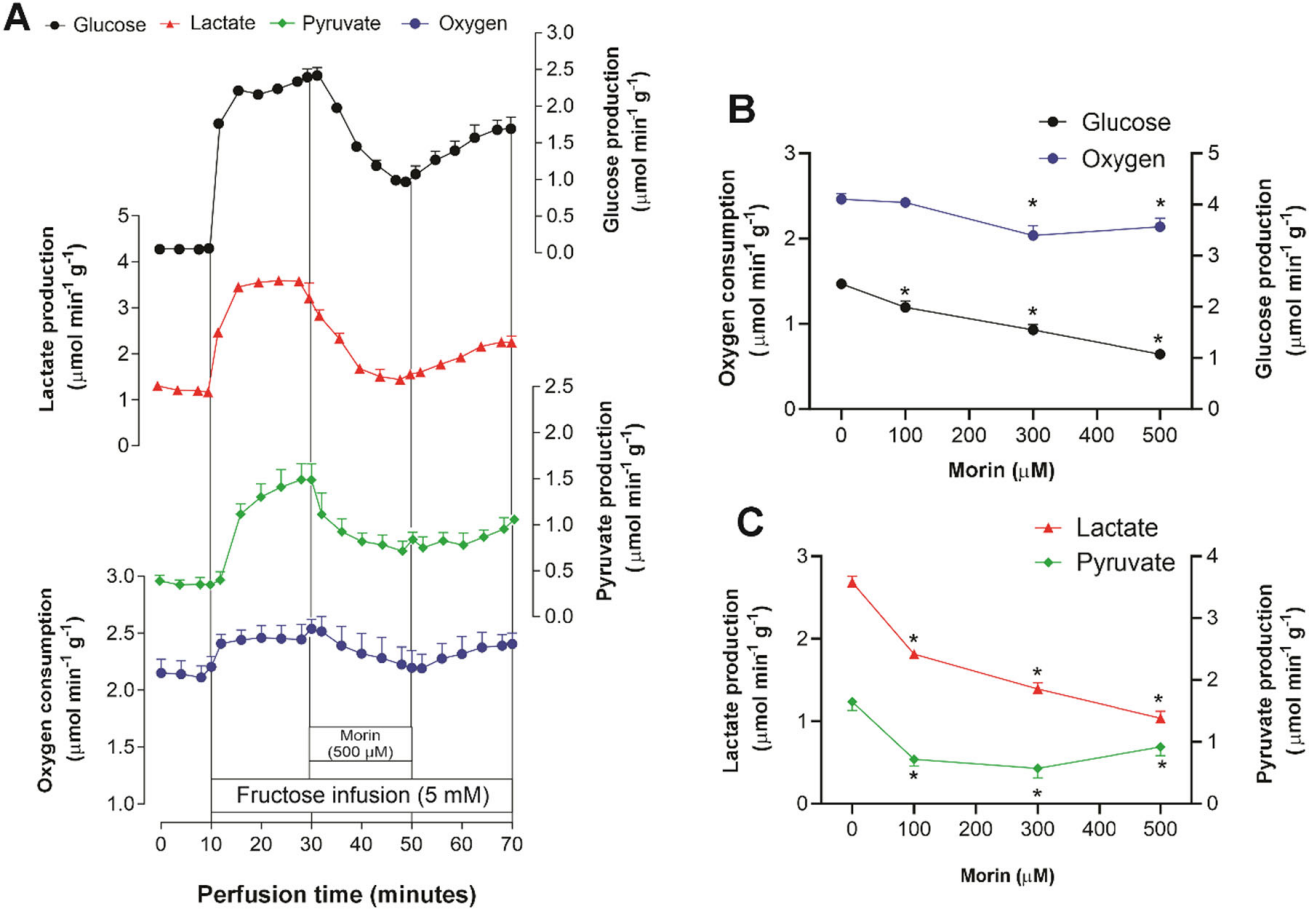
Effects of morin on fructose metabolic fluxes in isolated perfused livers from fasted rats. (A) Temporal changes induced by 500 μM morin on glucose production, lactate and pyruvate production, and oxygen consumption. (B) Concentration‐dependent effects of morin on glucose production and oxygen consumption. (C) Concentration‐dependent effects of morin on lactate and pyruvate production. Each experimental point represents the mean of four independent experiments, with bars showing standard errors of the mean. *p < 0.05, ANOVA with Dunnett's post hoc test.

The direct effects of varying concentrations of morin (100, 300, and 500 μM) on fructose metabolism under gluconeogenic conditions are illustrated in Figure 2. Figure 2A depicts the effects of 500 μM morin on the different parameters assessed in this experimental setup. Morin significantly inhibited oxygen consumption at concentrations of 300 and 500 μM (Figure 2B). Glucose production was notably suppressed at all concentrations, with a maximum inhibition of 56.15% at 500 μM (Figure 2B). The presence of fructose stimulated the production of lactate and pyruvate. However, morin infusion led to a significant reduction in both metabolites. Lactate production decreased in a concentration‐dependent manner, while pyruvate production exhibited a distinct inhibition pattern, with saturation starting from 100 μM morin (Figure 2C).

### 3.4 The Effects of Morin on Gluconeogenesis From Alternative Substrates

Experiments similar to those conducted with lactate, pyruvate, and fructose were repeated using three other gluconeogenic substrates: alanine (5 mM), dihydroxyacetone (2 mM), and glycerol (2 mM), with and without the infusion of 300 μM morin. The results are summarized in Table 1. Morin did not significantly inhibit gluconeogenesis from dihydroxyacetone. However, gluconeogenesis from alanine and glycerol was inhibited by 30.02% and 58.03%, respectively, in the presence of morin. In comparison, gluconeogenesis from lactate and pyruvate was inhibited by approximately 60% with 300 μM morin. For the exogenous alanine and glycerol, oxygen consumption was reduced by 13.77% and 16.71%, respectively. When dihydroxyacetone was used as a substrate, oxygen consumption was not significantly altered by morin.

**Table 1:** Effects of morin on gluconeogenesis from various substrates and related metabolic fluxes. The experimental protocols were analogous to those illustrated in Figures 1 and 2, with a final morin concentration of 300 μM. Values recorded in the absence of morin (control values) represent the averages immediately before the start of morin infusion. Values obtained in the presence of morin were calculated at the end of the infusion period.

Inflowing perfusate conditions	Metabolite production or oxygen consumption (µmol × min^−1^ × g^−1^)					
	Glucose	Oxygen	Pyruvate	Lactate	Ammonia	Urea
Alanine (5 mM)	1.179 ± 0.047^a^	2.817 ± 0.053^b^	0.526 ± 0.187	1.466 ± 0.099^c^	0.494 ± 0.072^d^	0.302 ± 0.008^e^
Alanine (5 mM) + Morin (300 µM)	0.825 ± 0.021^a^	2.429 ± 0.122^b^	0.805 ± 0.059	1.104 ± 0.098^c^	0.219 ± 0.063^d^	0.107 ± 0.021^e^
Dihydroxyacetone (2 mM)	1.082 ± 0.059	2.312 ± 0.109	0.581 ± 0.060	1.240 ± 0.161	‐‐	‐‐
Dihydroxyacetone (2 mM) + Morin (300 µM)	0.957 ± 0.057	2.073 ± 0.136	0.521 ± 0.061	0.768 ± 0.150	—	—
Glycerol (2 mM)	1.097 ± 0.068^f^	2.298 ± 0.115^g^	0.241 ± 0.060	0.921 ± 0.036	—	—
Glycerol (2 mM) + Morin (300 µM)	0.460 ± 0.080^f^	1.914 ± 0.046^g^	0.414 ± 0.080	0.765 ± 0.047	—	—

*Note:* The data are expressed as means ± standard errors of the mean from three to four experiments. Identical superscripts indicate significant differences between values with morin and the corresponding control conditions, as assessed by Student's *t*‐test (*p* < 0.05).

In addition to being converted into glucose in the liver, alanine, dihydroxyacetone, and glycerol can contribute to the formation of lactate and pyruvate. The effects of morin on the synthesis of these metabolites were also examined. While trends were observed, morin did not significantly affect lactate or pyruvate production when dihydroxyacetone and glycerol were used as substrates. When alanine was the gluconeogenic substrate, morin infusion inhibited lactate production but had no significant effect on pyruvate production.

Experiments with exogenous alanine as substrate also allowed the investigation of morin's effects on urea production, an energy‐dependent biosynthetic process. As shown in Table 1, ammonia and urea production were inhibited by 55.66% and 64.56%, respectively.

### 3.5 The Effects of Morin on Glycogenolysis and Glycolysis From Endogenous Glycogen, as Well as Oxygen Consumption, in the Liver of Fed Rats

**Figure 3:**
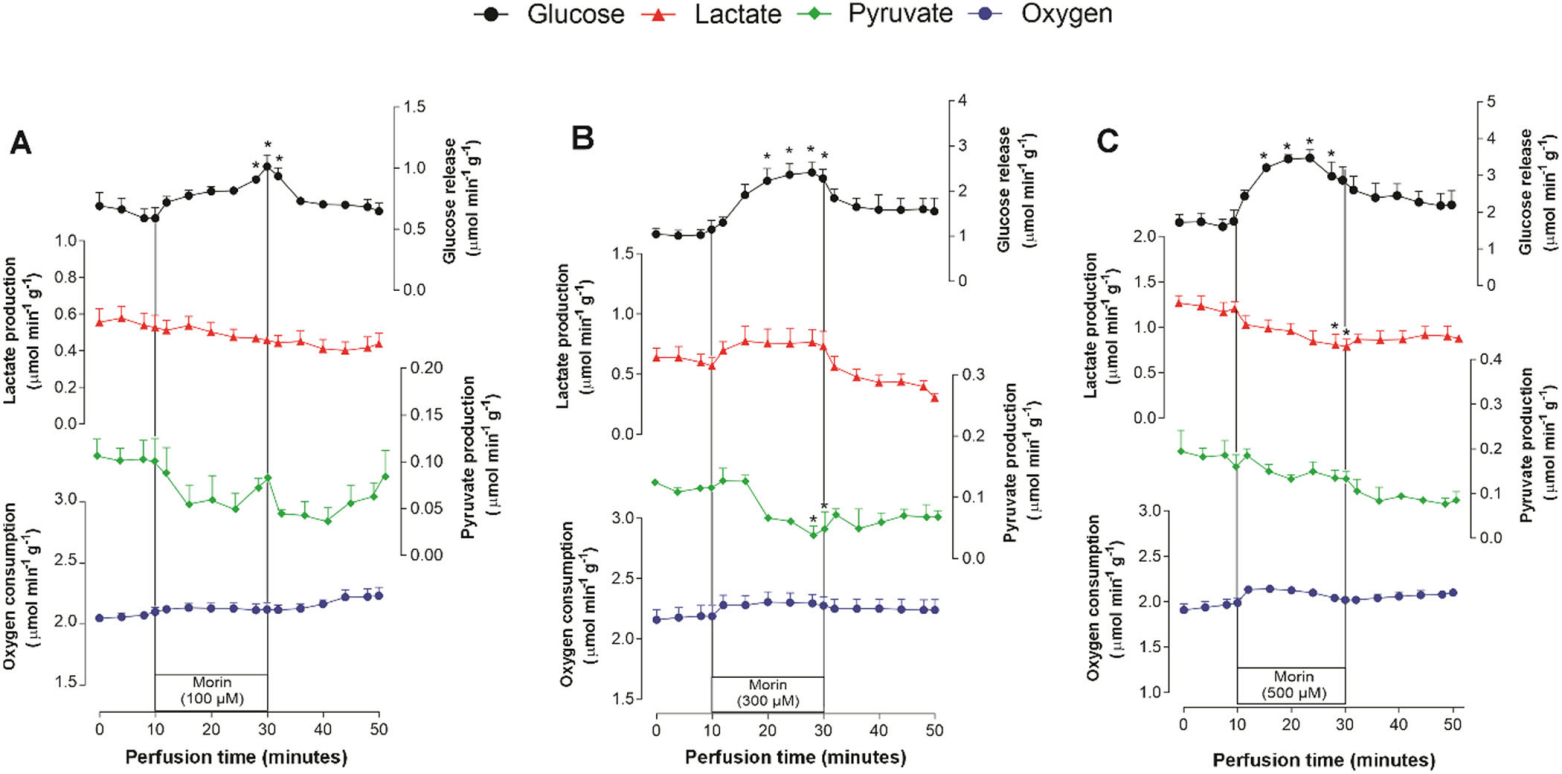
Time courses of the effects induced by 100–500 μM morin (A–C) on glycogen catabolism and oxygen uptake. Livers from fed rats were perfused with a substrate‐free medium. Morin was infused at concentrations of 100–500 μM during the period from 10 to 30 min, as indicated by the horizontal bars. Each data point represents the mean ± standard error of the mean from three to five experiments. Asterisks denote statistical significance compared to the control condition (values measured immediately before morin infusion), as determined by ANOVA followed by Dunnett's post hoc test (*p* < 0.05).

The effects of morin infusion at concentrations ranging from 100 to 500 μM on the perfused livers of fed rats are illustrated in Figure 3A–C. Before morin infusion, the values remained relatively stable. There was a noticeable stimulation in glucose release upon morin infusion (100–500 μM). However, while oxygen consumption showed a trend toward stimulation, the increase was not statistically significant when comparing values recorded in the presence of morin to those measured before the infusion. For glucose release, partial reversal was observed once the morin infusion was discontinued, but this effect was less apparent for oxygen consumption.

The responses of lactate and pyruvate production to morin infusion were more complex and depended on the flavonol's concentration. At the lowest concentration (100 μM), morin weakly inhibited both lactate and pyruvate production, though the effects were not statistically significant. At 300 μM, morin appeared to stimulate lactate production while inhibiting pyruvate production. At the highest concentration tested (500 μM), lactate production was inhibited, whereas pyruvate production showed a tendency toward reduction. In some cases, partial reversals of these effects were observed after the discontinuation of morin infusion.

### 3.6 The Effects of Morin on Exogenous Glucose Catabolism (Glycolysis)

**Figure 4:**
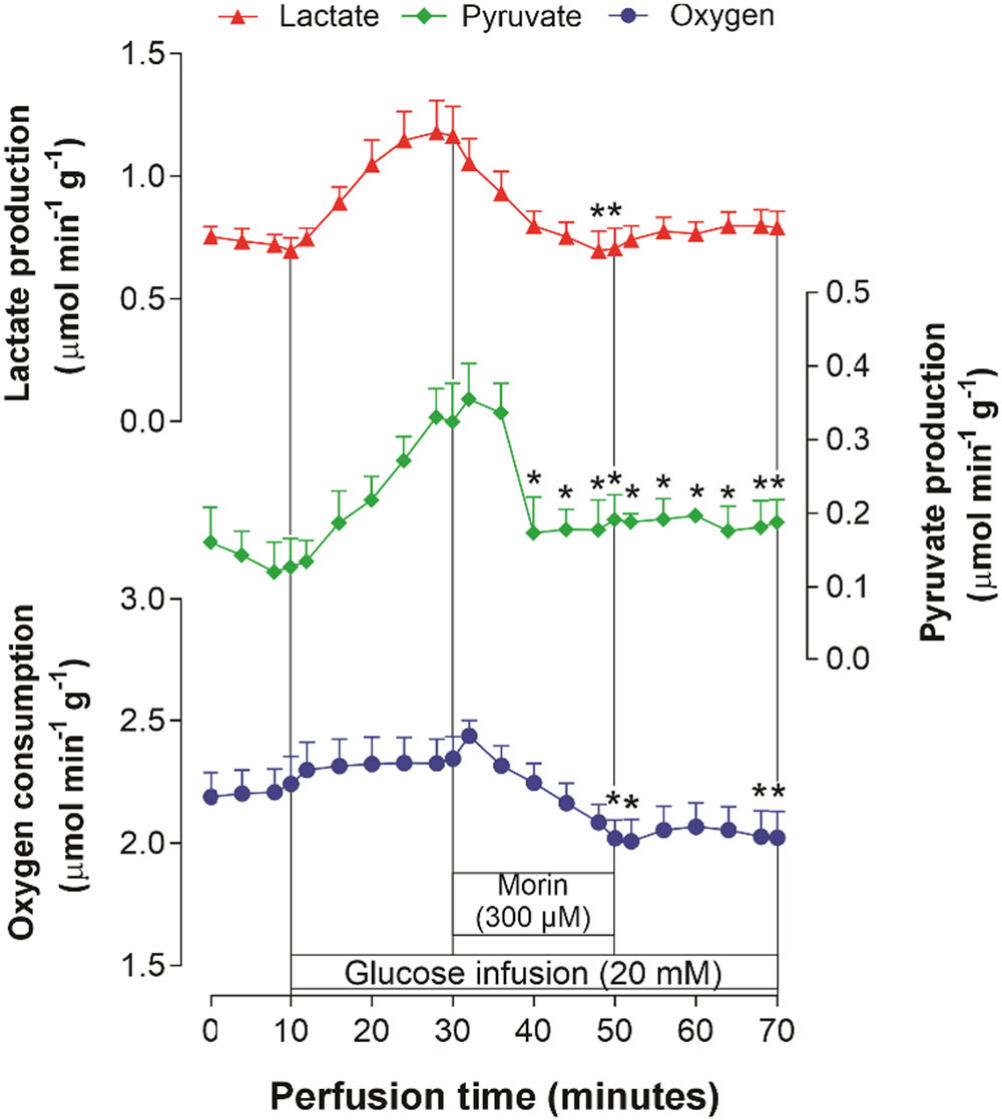
Rates of lactate and pyruvate production and oxygen consumption due to the infusion of exogenous glucose (20 mM) and the influence of 300 µM morin in perfused livers from fasted rats. Each data point represents the mean of four independent experiments, with error bars indicating the standard error of the mean. *p < 0.05, ANOVA with Dunnett's post hoc test.

As shown in Figure 4, under experimental conditions using livers from fasted rats, the infusion of exogenous glucose (20 mM) resulted in significant increases in lactate and pyruvate production, as well as oxygen consumption. Analysis of these three parameters revealed that the introduction of morin led to a considerable reduction in all measures, with marked decreases observed towards the end of the 300 μM morin infusion. Specifically, the inhibition rates for oxygen consumption, lactate production, and pyruvate production were 17.7%, 39.3%, and 40.7%, respectively. After discontinuing the morin infusion, lactate production showed a tendency to return to baseline levels, while oxygen consumption and pyruvate production remained significantly suppressed.

### 3.7 The Effects of Morin on Adenine Nucleotide Levels in Perfused Livers of Rats Under Fasted and Fed Conditions

Several effects of morin in liver perfusion experiments were associated with either inhibition or stimulation of oxygen consumption, suggesting that mitochondrial energy transduction systems might be influenced, potentially impacting cellular ATP production. To explore this, adenine nucleotide levels (AMP, ADP, and ATP) were quantified under gluconeogenic and glycogenolytic conditions (Tables 2 and 3). To obtain a more integrated view of cellular energy status, we also calculated the ATP/ADP and ATP/AMP ratios. These ratios are key indicators of hepatocellular energy charge and provide a more sensitive reflection of mitochondrial functionality than absolute nucleotide levels. The ATP/AMP ratio, for example, is particularly responsive to shifts in energy availability and can modulate some enzyme activities. In our model, the significant reductions in these ratios observed under morin treatment suggest a disruption in mitochondrial ATP synthesis and an increased activation of energy‐sensing pathways. Similar approaches using these ratios have been validated in studies with carbenoxolone, silibinin, metformin, and BHA in perfused livers [66, 67, 68, 69]. In the livers of fasted rats, morin presence led to a significant 40% reduction in ATP levels, while ADP and AMP levels did not show statistically significant changes. Additionally, the ATP/ADP ratio decreased by 36.17%, and the ATP/AMP ratio by 69.49%. In contrast, in the livers of fed rats, ATP, ADP, and AMP levels, as well as the ATP/ADP ratio, were not significantly affected by morin. However, the ATP/AMP ratio showed a significant decrease of 62.23%.

**Table 2:** Effects of morin on hepatic adenine nucleotide content in perfused livers of fasted rats. The livers of fasted rats were perfused in an open system, as described in the Materials and methods section. Lactate (2.0 mM) and pyruvate (0.2 mM) were infused at 10 min, and 300 μM of morin was infused 20 min after the initiation of lactate and pyruvate infusion. The livers were snap‐frozen in liquid nitrogen and extracted with perchloric acid. Neutralized extracts were used for the determination of adenine nucleotide content via HPLC. Control measurements were conducted on livers that were frozen at the same perfusion time but without morin infusion.

	µmol × (g liver weight)^−1^				
Conditions	ATP	ADP	AMP	ATP/ADP ratio	ATP/AMP ratio
Control (*n* = 4)	1.15 ± 0.20^a^	0.49 ± 0.02	0.23 ± 0.06	2.35 ± 0.31^b^	6.95 ± 1.30^c^
Morin (300 µM) (*n* = 4)	0.69 ± 0.07^a^	0.46 ± 0.04	0.31 ± 0.03	1.50 ± 0.13^b^	2.12 ± 0.51^c^

*Note:* The data are presented as means ± standard errors. Identical superscripts indicate significant differences between the values in the presence of morin and their respective control conditions, as revealed by Student's *t*‐test (*p* < 0.05).

**Table 3:** Effects of morin on hepatic adenine nucleotide content in perfused livers of fed rats. The livers of fed rats were perfused in an open system, as described in the Materials and methods section. The Krebs‐Henseleit buffer was infused for 10 min and, after that, 300 μM of morin was infused for 20 min. The livers were snap‐frozen in liquid nitrogen and extracted with perchloric acid. The neutralized extracts were used for the determination of adenine nucleotide content via HPLC. Control measurements were conducted on livers that were frozen at the same perfusion time but without morin infusion.

	µmol × (g liver weight)^−1^				
Conditions	ATP	ADP	AMP	ATP/ADP ratio	ATP/AMP ratio
Control (*n* = 5)	0.61 ± 0.13	0.27 ± 0.07	0.20 ± 0.06	2.82 ± 0.64	5.11 ± 1.09^a^
Morin (300 µM) (*n* = 5)	0.53 ± 0.10	0.25 ± 0.02	0.22 ± 0.03	2.00 ± 0.18	1.93 ± 0.82^a^

*Note:* The data are presented as means ± standard errors. Identical superscripts indicate significant differences between the values in the presence of morin and their respective control conditions, as revealed by Student's *t*‐test (*p* < 0.05).

### 3.8 The Effects of Morin on Oxygen Uptake by Perfused Livers

**Figure 5:**
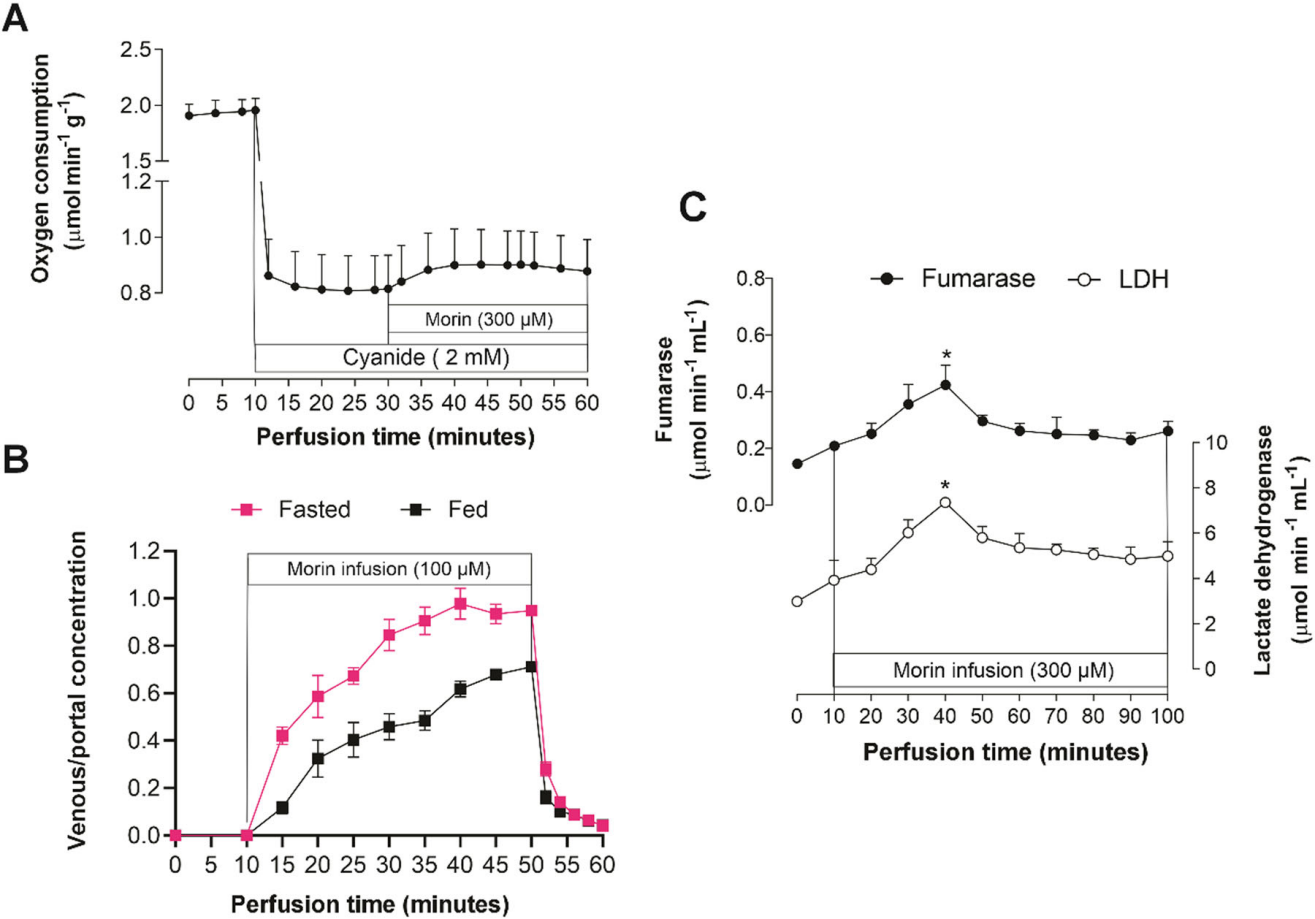
(A) Changes in hepatic oxygen consumption induced by morin in the presence of 2 mM cyanide. Livers were perfused in an open system as described in the Materials and Methods section, using a substrate‐free perfusion medium. Cyanide (2 mM) was infused for 50 min, followed by a 30‐min infusion of morin. (B) Normalized outflow profiles of morin. Livers from fasted or fed rats (as indicated in the legend) were perfused with Krebs‐Henseleit bicarbonate buffer, pH 7.4. Morin (100 µM) was infused for 40 min (10–50 min), as indicated by the horizontal bars. Morin was detected by HPLC. Venous‐to‐portal concentration ratios were plotted against infusion time. (C) Time course of fumarase and lactate dehydrogenase release in the perfusate induced by morin (300 μM) infusion. Livers from fed rats were perfused as described in the Materials and Methods section. Each experimental point represents the mean of three to five independent experiments, with error bars representing the standard error of the mean. *p < 0.05, ANOVA with Dunnett's post hoc test.

In certain experiments, particularly those examining the effects of morin on glycogenolysis and related parameters, the flavonol appeared to stimulate oxygen consumption, though this effect did not reach statistical significance. To determine whether the stimulation of oxygen consumption was exclusively due to mitochondrial activity, experiments were conducted using cyanide at a concentration of 2 mM, which effectively inhibits the mitochondrial respiratory chain (Figure 5A) [61]. Under these conditions, the liver's respiratory rate decreased from 1.95 ± 0.18 to 0.81 ± 0.12 μmol/min × g, with the latter value reflecting the combined activity of the microsomal electron transport chain and oxygen diffusion loss. Even under these conditions, 300 μM morin still showed a tendency to increase oxygen consumption, suggesting that the microsomal electron transport chain partially contributes to the morin‐induced rise in oxygen consumption.

### 3.9 Evaluation of Outflow Profiles and Morin Biotransformation

The results from the previous experiments (Figure 5A) suggest that the biotransformation of morin may be responsible for the stimulation of microsomal respiration. Additionally, studies have shown that morin undergoes conjugation reactions, including glucuronidation, sulfation, and methylation, in colonocytes and the liver. These processes are mediated by UDP‐glucuronosyltransferases, sulfotransferases, and catechol‐*O*‐methyltransferases [19]. In the liver, glucuronic acid is primarily derived from glycogenolysis. However, in fasted rats, where glycogen levels are minimal, gluconeogenesis becomes the exclusive source of glucuronic acid [61]. Consequently, it is plausible that the diversion of glucose 6‐phosphate for glucuronidation reactions could be a contributing factor to the inhibition of gluconeogenesis and glycolysis. To explore this possibility, efforts were made to verify the intracellular production of glucuronide metabolites.

Quantitative analysis is facilitated by evaluating the mean normalized outflow profiles of morin. Normalization was achieved by dividing the concentration of morin in the venous perfusate by the corresponding concentration in the portal perfusate. This normalization process facilitates comparisons across different conditions (e.g., livers from fed *vs.* fasted rats) and supports quantitative analysis [70]. As illustrated in Figure 5B, the venous‐to‐portal ratios of morin reached or approached steady‐state values below unity, which likely reflects its metabolic biotransformation and/or binding to intracellular structures such as hepatocyte organelles [70]. When morin was infused into livers from fasted rats, the transformation fraction was approximately 5% after 40 min of perfusion. In contrast, in livers from fed rats, this fraction increased markedly to around 30% after the same perfusion time. Additionally, the outputs of morin were delayed following the cessation of infusion.

### 3.10 The Effects of Morin on Enzyme Release in the Perfused Liver

Disruptions in enzyme levels, which may impact gluconeogenesis, frequently serve as markers of alterations in membrane fluidity, trafficking, and permeability [61]. To examine this potential link, the current study measured the release of two enzymes: lactate dehydrogenase, mainly found in the cytosol, and fumarase, primarily located in mitochondria. The outcomes of these measurements are depicted in Figure 5C. Livers from fed rats were infused with 300 μM morin for 90 min, and the activities of LDH and FUM were assessed in the effluent perfusate. Both enzymes demonstrated markedly rapidly release kinetics in the presence of morin. After 30 min of infusion, morin significantly increased LDH and FUM release by 87.54% and 103.45%, respectively. However, beyond this point, the release kinetics of these enzymes plateaued, so that in subsequent collection times, although enzyme activities were higher compared to the control (absence of morin, up to 10 min of perfusion), the differences were not statistically significant.

### 3.11 The Effects of Morin on Enzymatic Activities

Some of the observed effects of morin on gluconeogenesis and glycolysis could be attributed to the inhibition of key metabolic enzymes. Consequently, the effects of morin on the activities of glucose 6‐phosphatase, pyruvate kinase, glucokinase, and fructokinase were systematically evaluated. Additionally, the activity of glucose 6‐phosphate dehydrogenase, the first enzyme of the pentose phosphate pathway, was also assessed.

**Figure 6:**
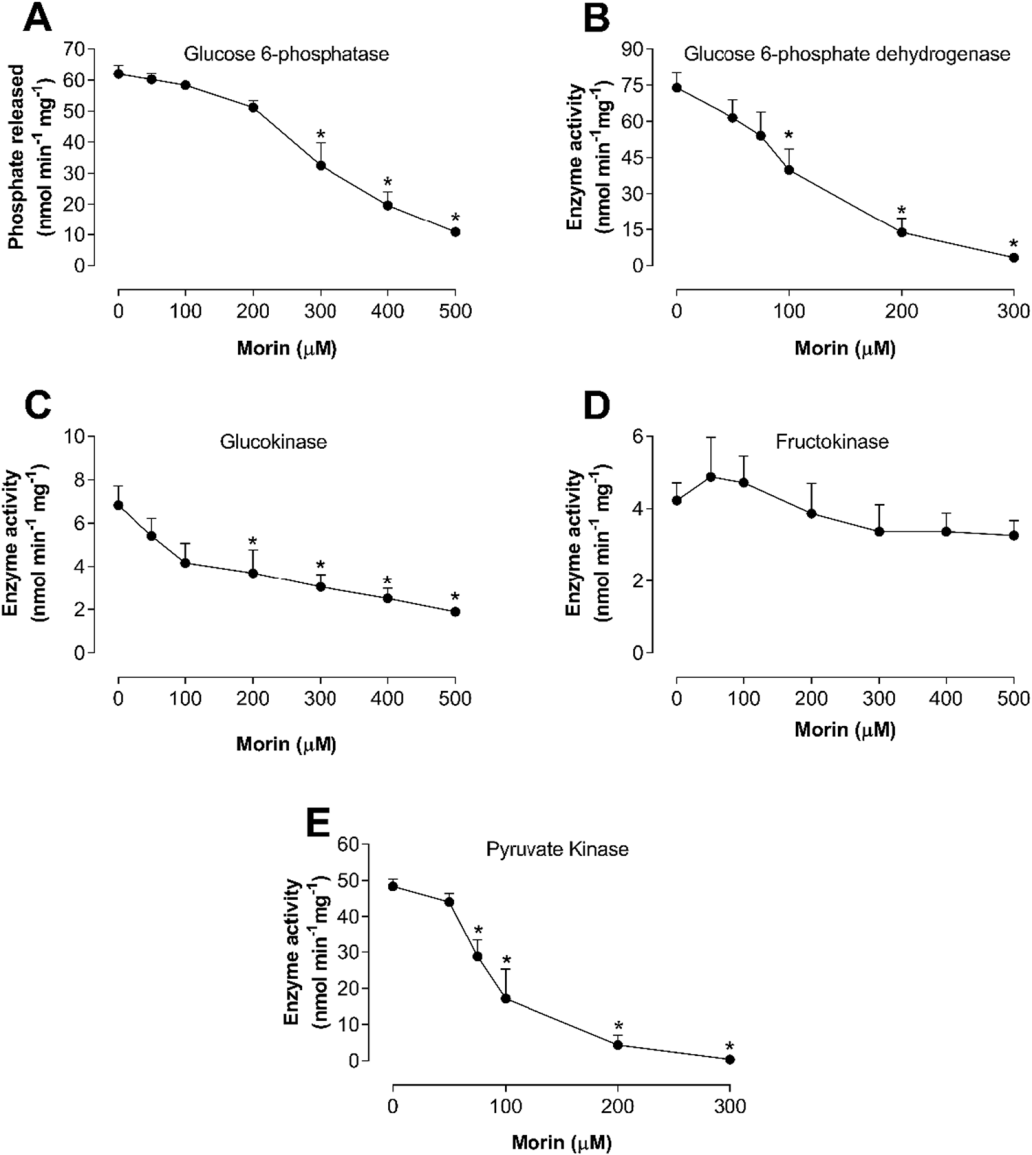
(A) activity of glucose 6‐phosphatase. (B) Activity of glucose 6‐phosphate dehydrogenase. (C) Activity of glucokinase. (D) activity of fructokinase. (E) Activity of pyruvate kinase. The enzymatic kinetics were analyzed as described in the Materials and Methods section. Morin was used at various concentrations (25–500 µM or 25–300 µM). Each experimental point represents the mean of 4 independent experiments, and the error bars indicate the standard error of the mean. *p < 0.05, ANOVA with Dunnett's post hoc test.

As illustrated in Figure 6A–E, morin clearly inhibited the activities of glucose 6‐phosphatase, glucose 6‐phosphate dehydrogenase, pyruvate kinase, and glucokinase in a concentration‐dependent manner. However, it did not significantly affect fructokinase activity. The maximum inhibition observed was 82.47% for glucose 6‐phosphatase (500 μM), 72.2% for glucokinase (500 μM), 99.2% for pyruvate kinase (300 μM), and 95.6% for glucose 6‐phosphate dehydrogenase (300 μM).

### 3.12 The Effects of Morin on Bioenergetics‐Related Parameters in Isolated Mitochondria

**Figure 7:**
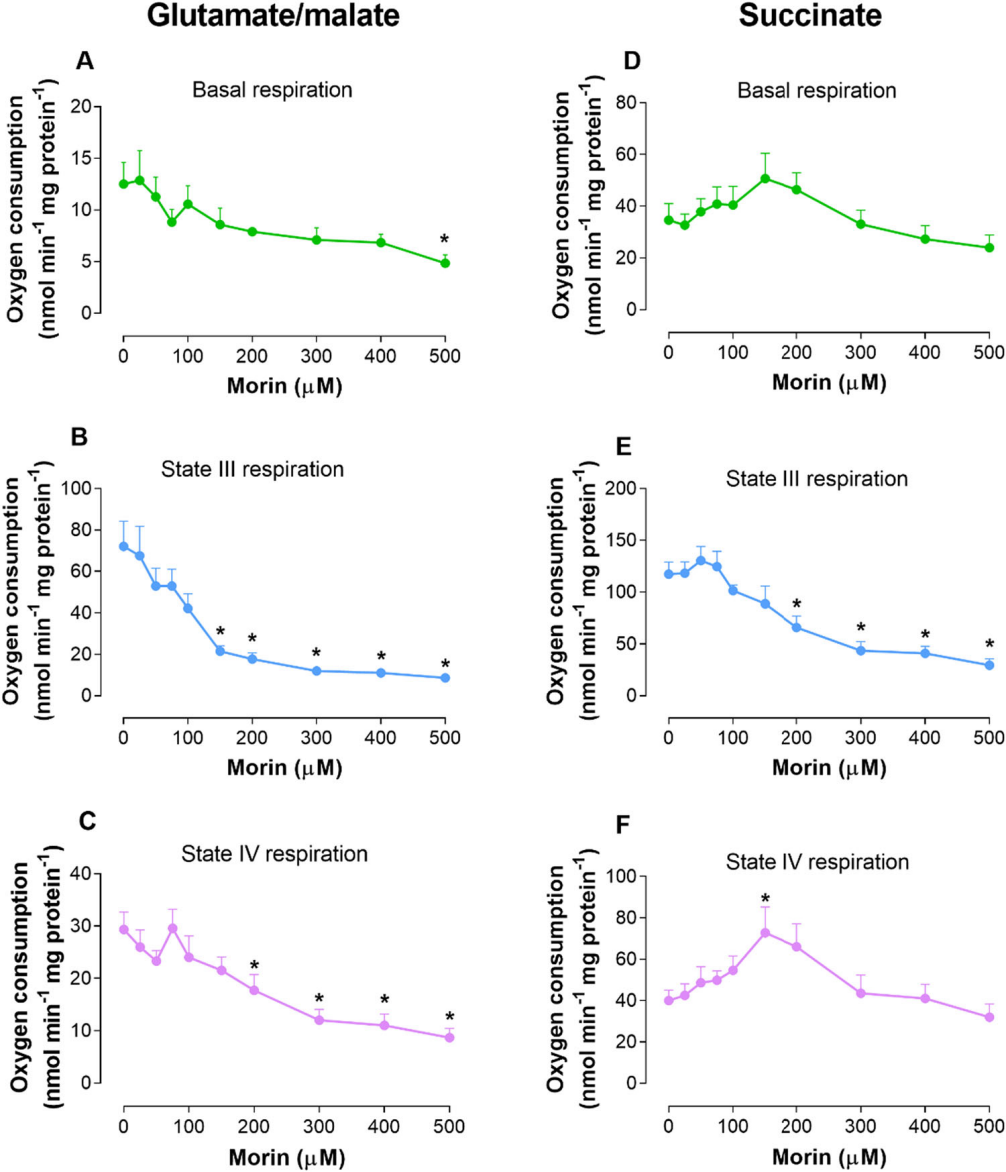
The effects of morin on mitochondrial respiration driven by a mixture of glutamate and malate (A–C) and succinate (D–F) with or without exogenously added ADP. Mitochondria were isolated and tested as described in the Materials and Methods section. Oxygen consumption rates were calculated from the slopes of the polarographic recordings. Each experimental point represents the mean of seven independent experiments, with error bars indicating the standard error of the mean. *p < 0.05, ANOVA with Dunnett's post hoc test.

Experiments with isolated mitochondria were conducted to complement earlier studies [37] and to aid in interpreting the data obtained from the perfused liver. The results indicate that morin has deleterious effects on mitochondrial respiration (Figure 7A–F and Table 4). In intact mitochondria energized with glutamate and malate, morin significantly inhibited both basal respiration and state IV respiration at elevated concentrations, showing inhibitions of 61.25% and 70.45%, respectively, at 500 μM. In contrast, morin tended to stimulate both basal respiration and, more notably, state IV respiration when respiration was supported by succinate, with the stimulation of state IV respiration peaking at 81.85% with 150 μM of morin. Nonetheless, at higher morin concentrations, a combined inhibitory effect emerged, overshadowing the initial stimulatory response. In the presence of exogenous ADP (state III respiration), significant inhibition was observed starting from 150 μM with glutamate and malate, and from 200 μM with succinate, reaching maximum inhibitions of 87.95% and 74.9%, respectively, at 500 μM. Table 4 presents the results regarding the effects of morin on the respiratory control ratio (RC) and the ADP/O ratio. For all substrates (glutamate and malate, as well as succinate), there was a reduction in the RC, primarily due to morin's effects on state III mitochondrial respiration. In the presence of glutamate and malate, mitochondria completely lost respiratory control starting from a morin concentration of 150 μM. A similar effect was observed with succinate. Although the ADP/O ratios were not significantly altered when morin was used at concentrations up to 100 μM, at higher concentrations, the effects of morin were so pronounced that the typical polarographic assay technique for determining ADP/O ratios could not be performed.

**Table 4:** The effects of morin on mitochondrial respiration driven by glutamate plus malate and succinate, both in the presence and absence of exogenously added ADP. Mitochondria were isolated and quantified as described in the Section 2. The respiratory control ratio (RC) and ADP/O ratio were calculated according to the method outlined by Chance and Williams [71].

Substrate	Morin (μM)	Respiratory control ratio (RC)	ADP/O ratio
Glutamate and malate	0	2.650 ± 0.420	2.444 ± 0.097
	25	2.753 ± 0.499	2.420 ± 0.114
	50	2.200 ± 0.235	2.340 ± 0.111
	75	1.808 ± 0.276	2.580 ± 0.271
	100	1.798 ± 0.188	2.494 ± 0.097
	150	1.00 ± 0.00^a^	—*
	200	1.00 ± 0.00^a^	—*
	300	1.00 ± 0.00^a^	—*
	400	1.00 ± 0.00^a^	—*
	500	1.00 ± 0.00^a^	—*
Succinate	0	2.443 ± 0.438	1.614 ± 0.091
	25	2.681 ± 0.621	1.717 ± 0.157
	50	2.766 ± 0.489	1.533 ± 0.121
	75	2.381 ± 0.314	1.556 ± 0.071
	100	1.754 ± 0.155	1.385 ± 0.082
	150	1.00 ± 0.00^a^	—*
	200	1.00 ± 0.00^a^	—*
	300	1.00 ± 0.00^a^	—*
	400	1.00 ± 0.00^a^	—*
	500	1.00 ± 0.00^a^	—*

*Note:* The data represent the mean ± standard error of seven independent experiments conducted using an identical protocol. Statistical significance compared to the controls is indicated by asterisks.

^a^ Denotes an imprecise determination.

* *p *< 0.05, ANOVA with Dunnett's post hoc test.

**Figure 8:**
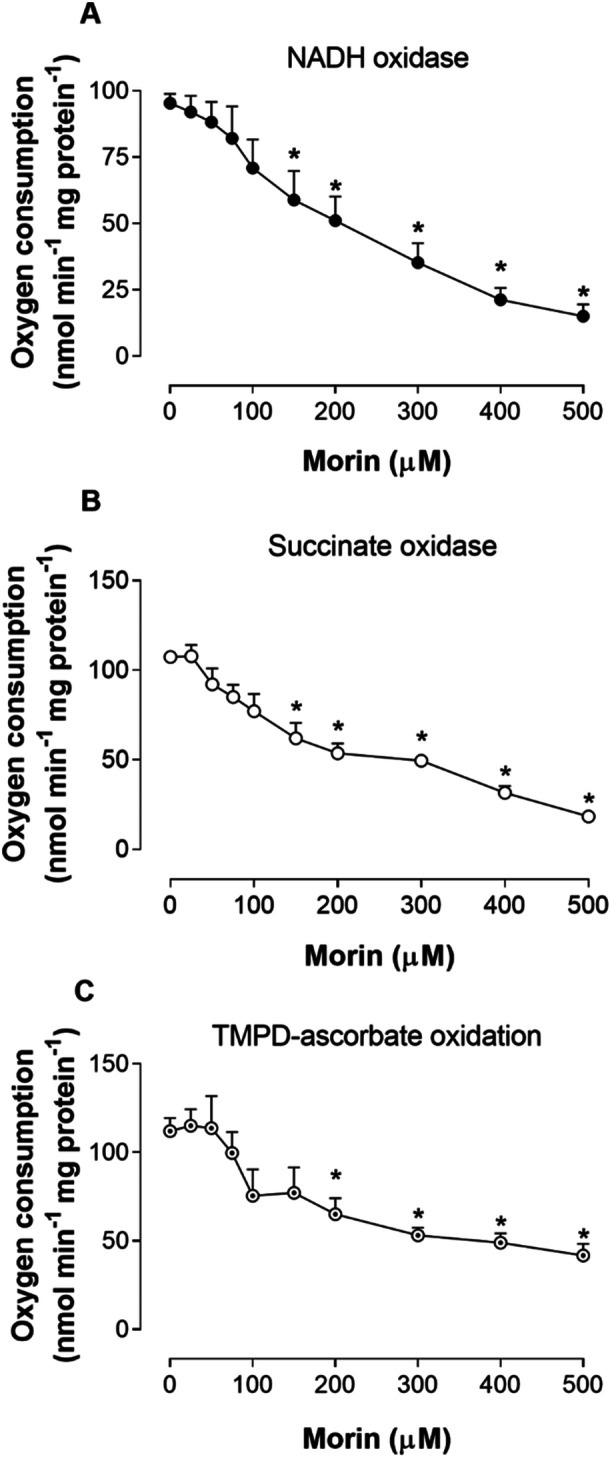
The effects of morin on various enzyme activities associated with mitochondrial membranes in rat liver. NADH oxidase (A), succinate oxidase (B), and TMPD‐ascorbate oxidation (C) were measured in mitochondria disrupted by freeze‐thaw cycles, incubated at 37°C in the reaction medium, as described in the Materials and Methods section. Each data point represents the mean of four to five independent experiments, with error bars indicating the standard error of the mean. *p < 0.05, ANOVA with Dunnett's post hoc test.

The differing impacts of morin on state IV and basal respiration with glutamate and malate versus succinate as substrates indicate that morin affects specific components of the respiratory chain that are not common to the oxidation of these substrates. To explore this hypothesis, we assessed respiratory activity in mitochondria disrupted by freeze‐thaw cycles, using NADH to evaluate NADH oxidase activity, succinate for succinate oxidase activity, and TMPD‐ascorbate for complex IV activity. The findings are illustrated in Figure 8A–C. Morin suppressed the activities of both NADH and succinate oxidases. Respiration supported by NADH was inhibited starting at 150 μM, with a maximum inhibition of 84.17% observed at 500 μM. Succinate‐driven respiration was similarly reduced by 82.92% at 500 μM of morin. Additionally, the flavonol exhibited effects on TMPD‐ascorbate oxidation, significantly decreasing activity from 200 μM onwards, and achieving a substantial inhibition of 62.71% at 500 μM of morin.

**Figure 9:**
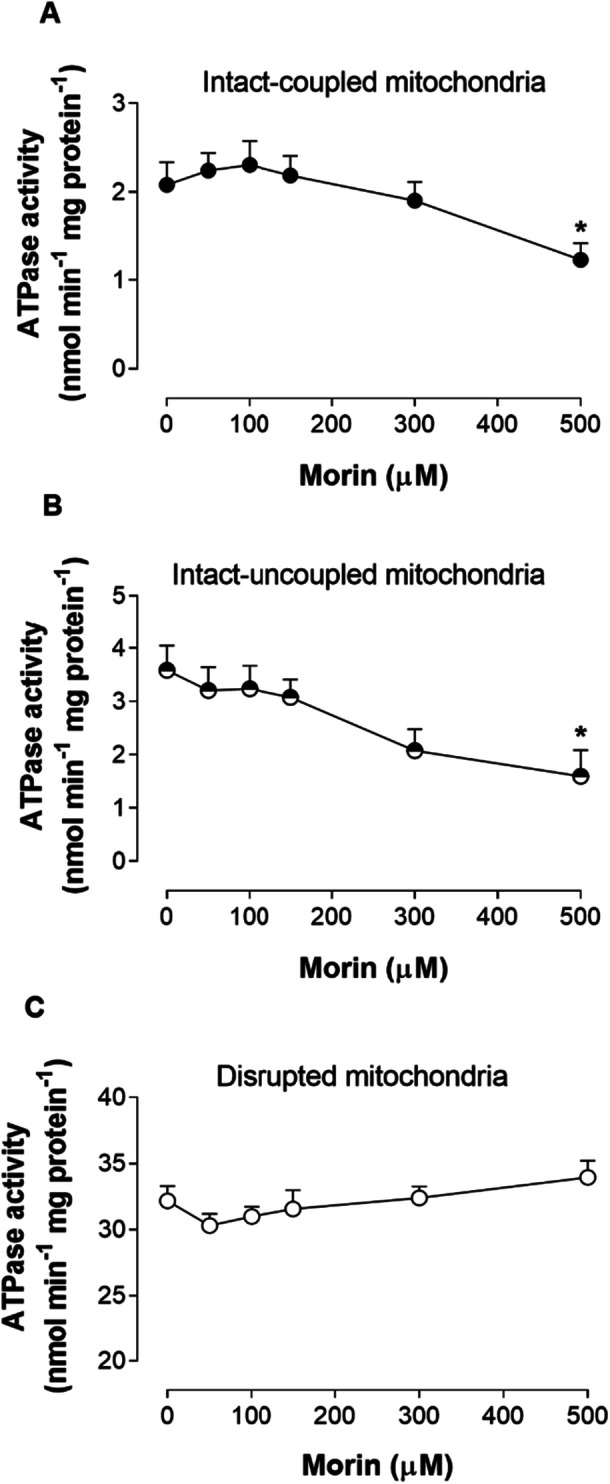
The effects of morin on ATPase activity in coupled mitochondria (A), uncoupled mitochondria (B), and freeze‐thaw‐disrupted mitochondria (C). Each data point represents the mean of 6 independent experiments, with error bars indicating the standard error of the mean. *p < 0.05, ANOVA with Dunnett's post hoc test.

The observed inhibition of state III respiration by morin might be attributable to a direct interaction with the F_o_F_1_–ATP synthase complex. To further investigate this potential mechanism, ATPase activity was measured across three different mitochondrial preparations: intact‐coupled, intact‐uncoupled, and freeze‐thaw‐disrupted mitochondria. Figure 9A–C illustrates that the effects of morin were influenced by both its concentration and the specific mitochondrial preparation utilized. Morin inhibited ATPase activity in both intact‐coupled and uncoupled mitochondria, with a significant decrease observed only at a concentration of 500 μM, resulting in reductions of 44.56% and 55.48%, respectively. At lower concentrations (25–100 μM), there was a slight trend toward stimulation in intact‐coupled mitochondria. ATPase activity in freeze‐thaw‐disrupted mitochondria was not significantly affected.

**Figure 10:**
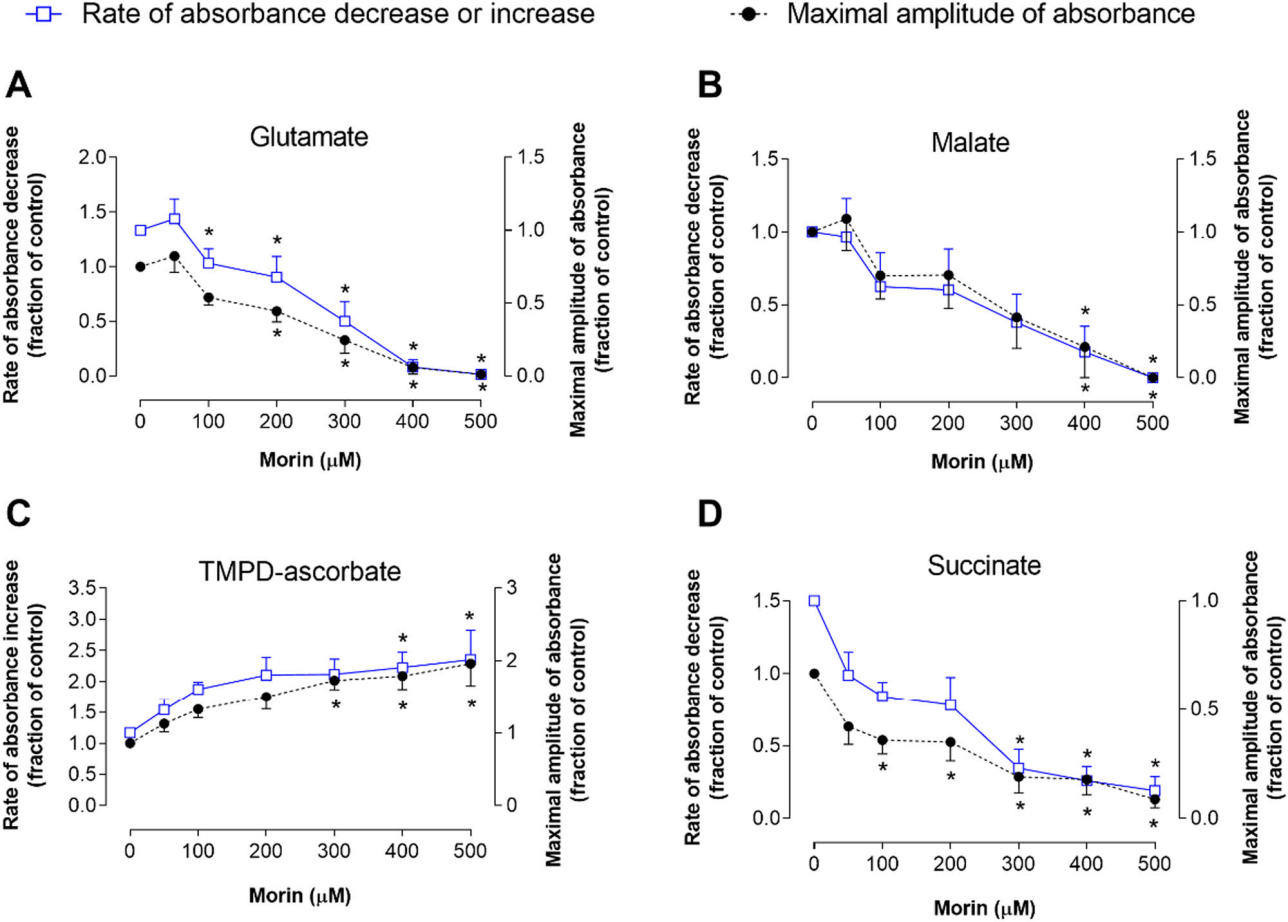
The effects of morin on mitochondrial swelling are depicted. Swelling reactions were initiated by the simultaneous addition of sodium acetate (50 mM) and the following substrates: (A) glutamate 10 mM; (B) malate 10 mM; (C) TMPD 0.2 mM with ascorbate 5 mM; and (D) succinate 10 mM. Changes in absorbance at 520 nm were recorded. The maximum absorbance amplitude and the rate of increase or decrease in absorbance were expressed as fractions of the corresponding controls and plotted against morin concentrations. Each data point represents the mean of four independent experiments, with error bars indicating the standard error of the mean. *p < 0.05, ANOVA with Dunnett's post hoc test.

To gain further insights into the mechanism of action of morin, we also investigated its effect on mitochondrial swelling. Mitochondrial volume changes, which are dependent on energy generated by the electron transport chain through organic substrate oxidation, were measured following the methodology outlined by Mustafa and colleagues [37]. Figure 10A–D presents the results from this series of experiments. When glutamate and malate were present as as oxidizable substrates, morin markedly inhibited mitochondrial swelling, particularly at higher concentrations. Morin affected both the initial rate and the total amplitude of swelling. In both parameters, 500 μM morin reduced swelling by approximately 98% for glutamate and 100% for malate. A significant decrease was also observed when succinate induced swelling, although the effect was less pronounced. Here, the rate of swelling was less affected, with a significant impact only at 300 μM of morin, while the amplitude of swelling was more notably reduced, reaching 86.94% at 500 μM. In contrast, in the presence of TMPD and ascorbate, morin caused a progressive increase in swelling. At 500 μM, the amplitude and rate of swelling reached their maximum increases of 102% and 128.9%, respectively.

**Figure 11:**
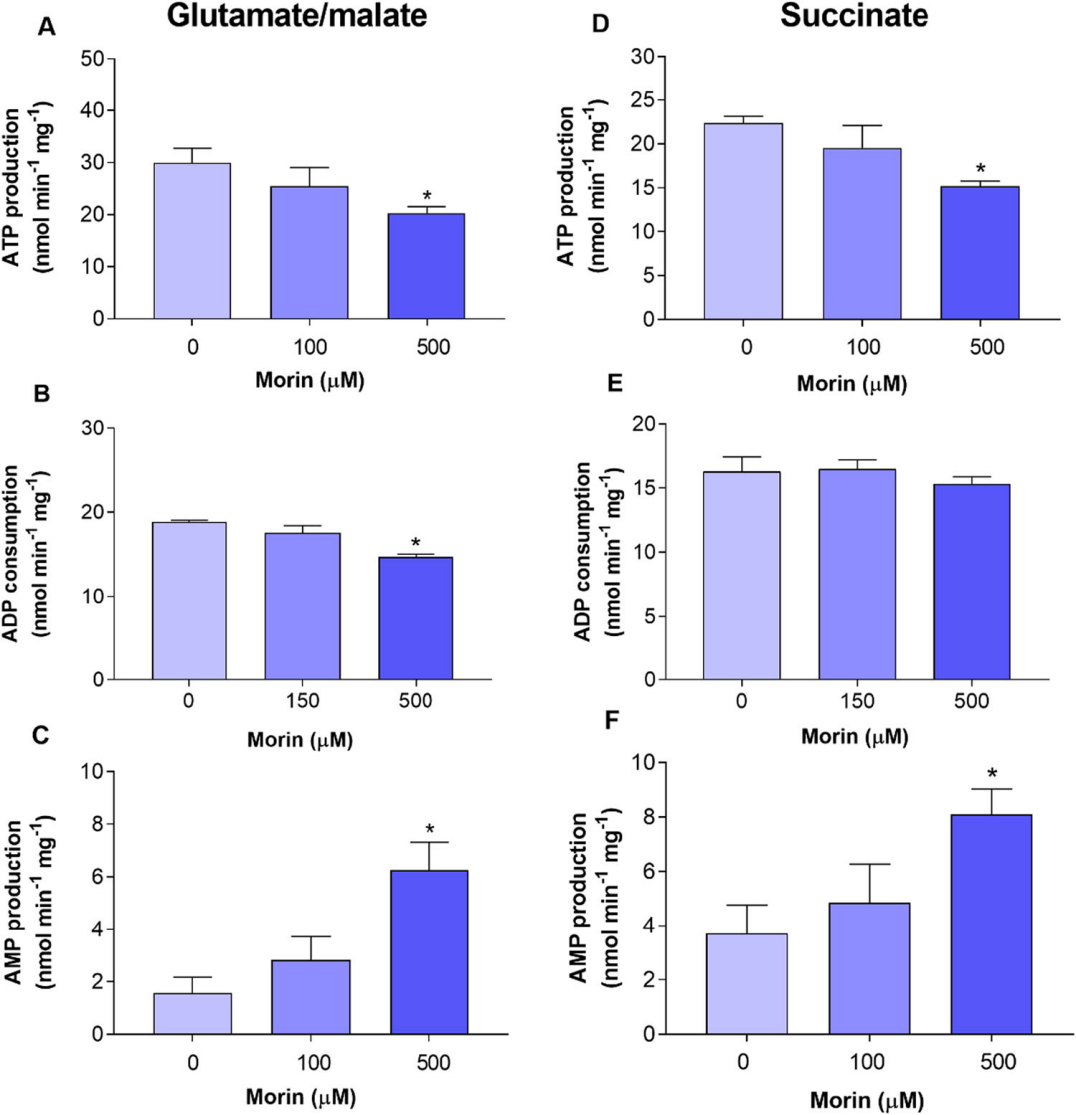
The effects of morin on ATP and AMP synthesis, as well as ADP consumption, in isolated rat liver mitochondria were assessed. The mitochondria were incubated as described in the Materials and Methods section in a reaction medium containing 10 mM glutamate plus 10 mM malate (A–C) or 10 mM succinate plus 5 μM rotenone (D–F). The levels of ATP, ADP, and AMP in the neutralized extract were determined by high‐performance liquid chromatography (HPLC). Each bar represents the mean of seven independent experiments, with error bars indicating the standard error of the mean. *p < 0.05, ANOVA with Dunnett's post hoc test.

These aforementioned results unequivocally demonstrate that morin exerts a multifaceted impact on mitochondrial oxidative metabolism. These effects are theoretically expected to result in the inhibition of oxidative phosphorylation. Figure 11A–F illustrates the ATP synthesis rate in isolated rat liver mitochondria in the presence of various substrates (succinate, malate, and glutamate). Overall, the inhibition exhibited a concentration‐dependent nature on ATP synthesis via oxidative phosphorylation, with significant inhibition observed at 500 μM compared to 100 μM morin. When glutamate and malate served as electron donors, the inhibition peaked at 32.46% with 500 μM morin. Comparatively, ATP synthesis was reduced by 32.11% when succinate was utilized as the electron donor. The inhibitory effect of morin on mitochondrial oxidative phosphorylation was further assessed by measuring ADP consumption and AMP production. In the presence of glutamate and malate, morin significantly inhibited ADP consumption at 500 μM. The use of succinate as the oxidizable substrate also reduced ADP consumption, albeit to a lesser extent, making the effect statistically insignificant. Although there was a trend towards AMP production stimulation at a concentration of 100 μM of morin, the results were not statistically significant at this level. The significant increase in AMP synthesis was observed only at 500 μM, where it reached a maximum of 300.89% in the presence of glutamate and malate, and 163.38% in the presence of succinate.

### 3.13 The Effects of Morin on the Cellular Viability of Vero and HepG2 Cells

**Figure 12:**
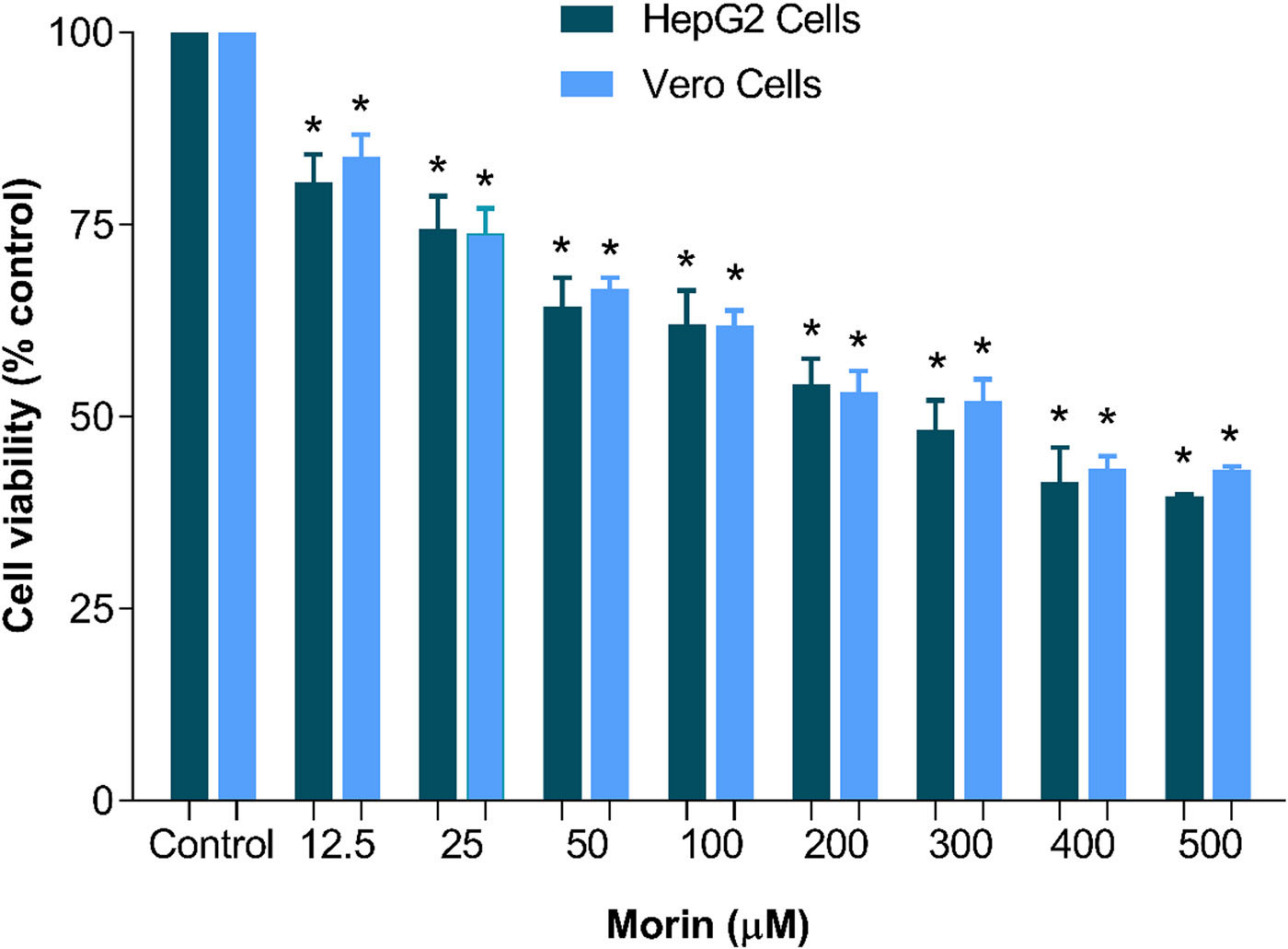
Effects of morin on the cellular viability of VERO and HepG2 cells. Each data point represents the mean of six independent experiments, with error bars indicating the standard error of the mean. *p < 0.05, ANOVA with Dunnett's post hoc test.

Considering morin's effects observed in liver perfusion experiments, particularly its ability to induce the release of cytosolic and mitochondrial enzymes, its deleterious impact on mitochondrial oxidative metabolism, and morin's documented capacity to suppress cell viability [34, 35, 36], we further explored its acute effects on the viability of hepatic cancer cells (HepG2) and renal epithelial cells (VERO), thereby extending and complementing previous research efforts. The cell viability of VERO and HepG2 cells was assessed following treatment with various concentrations of morin, as illustrated in Figure 12. Compared to the control condition, morin treatment exhibited a concentration‐dependent effect on cell viability, with viability dropping below 40% for HepG2 cells and below 45% for VERO cells at a concentration of 500 μM. The half‐maximal inhibitory concentration (IC_50_) was determined to be 208.34 μM for HepG2 cells and 225.52 μM for VERO cells.

## 4 Discussion

According to the findings of this study, morin can significantly influence hepatic gluconeogenesis and glycogenolysis, key metabolic pathways leading to hyperglycemia in type 2 diabetes [26, 27, 28, 29, 30]. The flavonol suppressed gluconeogenesis from multiple substrates in the livers of fasting animals and enhanced glycogenolysis in the livers of fed animals, providing partial support for the liver's acute role in morin's antihyperglycemic effects. These effects likely stem from its inhibition of mitochondrial energy transduction. Additionally, enzyme inhibition, biotransformation interference, and cellular disruptions—especially to membrane integrity—may contribute to altered metabolic fluxes in these liver pathways.

Morin's inhibitory effect on mitochondrial energy transduction involves at least three mechanisms: moderate uncoupling of oxidative phosphorylation, inhibition of the ATP/ADP exchange system, and suppression of the mitochondrial respiratory chain. Evidence of its uncoupling effect includes increased substrate‐driven and state IV respiration with succinate, reduced RC ratios in intact mitochondria, and inhibition of mitochondrial swelling with various substrates. Perfusion experiments, where morin tended to stimulate oxygen consumption—such as during glycogenolysis evaluation in fed rat livers—further support this. Morin may act as a protonophore, facilitating passive proton movement along the mitochondrial electrochemical gradient [66]. Additionally, its uncoupling may result from increased membrane permeability via direct interaction with the inner mitochondrial membrane [26]. This aligns with findings that morin promotes the release of cytosolic and mitochondrial enzymes in perfused rat liver. The suppression of ATPase activity in both intact‐coupled and intact‐uncoupled mitochondria, with little effect on freeze‐thaw disrupted mitochondria, suggests a weak inhibition of the ATP/ADP exchange system [66]. This is further supported by morin's inhibition of state III respiration in intact mitochondria during active phosphorylation.

The inhibition of NADH oxidase, succinate oxidase, and TMPD‐ascorbate oxidation in freeze‐thaw disrupted mitochondria indicates that morin also inhibits the mitochondrial respiratory chain. While current data do not allow a definitive conclusion on whether morin inhibits complex I, II, or both—or if it targets only complex III, thus affecting NADH and succinate oxidases—it is clear that morin inhibits complex IV, as shown by its suppression of TMPD‐ascorbate oxidation. The inhibition of both basal and state IV respiration with malate and glutamate, the peak of succinate‐driven state IV respiration at 150 μM morin followed by decline at higher doses, and the reduced mitochondrial swelling in the presence of malate, glutamate, and succinate further support morin's role as a mitochondrial respiratory chain inhibitor.

The complex effects of morin on mitochondria resulted in a reduction in ATP production capacity, especially at higher concentrations. Consequently, ADP consumption decreased or tended to decline. In contrast, AMP synthesis increased in isolated mitochondria, likely due to adenylate kinase activity, which catalyzes the reaction 2ADP ↔ ATP + AMP within mitochondria [72]. In intact liver, adenylate kinase may regulate gluconeogenic, glycolytic, and glycogenolytic enzymes via nucleotide exchange and AMP signaling, integrating these pathways to rapidly respond to energy demands and enhancing morin's impact on mitochondrial energy metabolism. Overall, morin increased glycogen breakdown while reducing gluconeogenesis, consistent with AMP signaling and metabolic regulation by adenylate kinase [72], and with decreased ATP/AMP ratios observed in livers of both fed and fasted rats. The reductions in ATP/ADP and ATP/AMP ratios observed in the presence of morin provide further support for mitochondrial dysfunction. These ratios serve not only as indicators of the energy charge but also act as metabolic sensors that influence regulatory networks involving some enzymes. A low ATP/AMP ratio is typically associated with activation of catabolic pathways and inhibition of biosynthetic fluxes like gluconeogenesis. Therefore, their decline under morin treatment is consistent with the observed metabolic alterations. This interpretation is supported by several studies that have applied similar analytical strategies in perfused rat liver models, including those evaluating the hepatic effects of carbenoxolone [66], diuron [73], BHA [69], and metformin [68].

Mitochondrial swelling assays serve as a sensitive indicator of changes in membrane permeability and mitochondrial bioenergetic function, particularly reflecting alterations induced by oxidative stress or membrane perturbations. The inhibition of swelling induced by glutamate, malate, and succinate supports morin's moderate uncoupling effect and its role as a respiratory chain inhibitor, as expected [59]. Therefore, morin's uncoupling action and its inhibition of TMPD‐ascorbate oxidation in freeze‐thaw disrupted mitochondria should theoretically prevent swelling in mitochondria stimulated by TMPD‐ascorbate [74]. However, this was not observed—morin instead induced swelling in the presence of TMPD‐ascorbate. This may result from the lipid composition of the inner mitochondrial membrane and morin's lipophilic core, which could allow its incorporation into the membrane and alter fluidity [75, 76]. Another plausible explanation is that morin induces mitochondrial permeability transition (MPT) by opening the permeability transition pore (PTP), a key event in mitochondrial apoptosis potentially linked to its uncoupling effect [77].

It is well known that, depending on the severity and type of mitochondrial dysfunction, cells may undergo necrotic death from energy depletion or activate apoptotic pathways due to metabolic stress [78, 79]. Consistent with this, experiments on VERO and HepG2 cells showed that morin acutely disrupts cellular integrity, leading to extensive cell death—supporting prior evidence of its apoptotic potential [34, 35, 36]. Morin may increase hepatic mitochondrial sensitivity to PTP opening through its moderate uncoupling effect, contributing to cytotoxicity. The fact that morin induces the release of cytosolic and mitochondrial enzymes suggests similar effects could occur in intact liver. The enzyme losses caused by morin likely extend beyond those measured here and should be seen as markers of cytosolic and mitochondrial protein release [61].

While enzyme losses are expected to reduce metabolic activity, it is noteworthy that morin‐induced release of lactate dehydrogenase and fumarase took about 30 min to reach significantly elevated levels in the perfusate. In contrast, metabolic changes like altered gluconeogenesis began almost immediately after morin infusion started. Thus, during the first 20 min—when metabolic effects were assessed—membrane damage or reduced cell viability were not the main factors influencing energy metabolism. Notably, only a transient, early increase in enzyme release from the intact liver was observed, which did not persist throughout the infusion. This may result from morin's biotransformation during its first liver passage, likely weakening its effects, especially those related to membrane disruption. As morin is metabolized into more water‐soluble forms, such as glucuronides [19], its lipophilicity decreases, reducing its ability to interact with and disrupt membranes. Still, prolonged use of high doses or accidental intake of large amounts may pose significant risks to liver function.

Regarding its effect on energy metabolism in perfused rat liver, morin acts similarly to several oxidative phosphorylation inhibitors [47, 73, 80, 81, 82]. Its stimulation of oxygen consumption in glycogenolytic states and inhibition under gluconeogenic conditions suggests a broader principle: moderate uncouplers like morin can also inhibit the electron transport chain [83], as shown in this study. The reduction in oxygen consumption during morin infusion aligns with its known inhibitory effects on the mitochondrial respiratory chain and ATP/ADP exchange system. Notably, oxygen consumption was not stimulated in the fasting state. Our results with trace amounts of [1–^14^C]palmitate support this finding, showing that high morin concentrations can impair the citric acid cycle under gluconeogenic conditions. This cycle is crucial for gluconeogenesis, especially in converting lactate and pyruvate [84, 85, 86]. Thus, reduced cycle activity—due to electron transport chain inhibition or direct enzymatic blockade—impairs gluconeogenesis [86]. Importantly, morin‐induced changes in gluconeogenesis from lactate and pyruvate, oxygen consumption, and citric acid cycle activity are interconnected. These effects likely result from disruptions in mitochondrial energy metabolism, including inhibition of NADH oxidase, succinate oxidase, cytochrome *c* oxidase (complex IV), and the ATP/ADP exchange system.

Beyond its detrimental effects on mitochondrial bioenergetics, some observations suggest alternative explanations. A key question concerns the lack of clear stimulation of glycolysis from endogenous glycogen, exogenous glucose, and in the presence of fructose, glycerol, and dihydroxyacetone—catabolic processes usually enhanced by decreased ATP availability, at least temporarily [47, 73, 80, 81, 82, 87]. Glycolysis inhibition may partly stem from suppressed glucose phosphorylation by glucokinase. However, this alone cannot explain the reduced glycolysis, as pathways from glycogen and other substrates like fructose, glycerol, and dihydroxyacetone bypass this step. Another likely factor is morin's inhibitory effect on pyruvate kinase, demonstrated in this study. Regarding fructose metabolism, inhibition of fructokinase seems unlikely, as morin showed no effect on this enzyme. However, the possibility that morin affects specific enzymatic stages in the metabolism of fructose, glycerol, and dihydroxyacetone cannot be completely excluded. For instance, triokinase, aldolase B, and fructokinase work together to metabolize fructose in the liver [88], offering several potential points for morin's action.

The extent of morin's effect on gluconeogenesis from substrates such as lactate, pyruvate, alanine, fructose, glycerol, and dihydroxyacetone cannot be fully explained by its impact on mitochondrial energy metabolism alone. Although the conversion of fructose, glycerol, and dihydroxyacetone into glucose is expected to be influenced by morin's effects on energy metabolism, glycolysis—an ATP‐generating pathway—occurs simultaneously, making their conversion less dependent on mitochondrial ATP than gluconeogenesis from lactate and alanine. Unexpectedly, glucose synthesis from glycerol was significantly inhibited by morin, suggesting interference in glycerol metabolism before its conversion into triose‐phosphates. A plausible explanation is the inhibition of glycerol kinase, which phosphorylates glycerol to glycerol 3‐phosphate. However, without direct experimental evidence, this remains speculative.

Alanine is another gluconeogenic substrate seemingly affected by mechanisms beyond mitochondrial energy disruption in the presence of morin. In perfused liver, alanine metabolism helps monitor ammonia and urea production. If a compound solely impairs oxidative phosphorylation, urea synthesis (an ATP‐dependent process) should decline while ammonia levels rise, given its toxicity. However, morin inhibited both ammonia and urea synthesis, suggesting that, beyond affecting mitochondrial bioenergetics, it may also inhibit enzymes directly involved in alanine metabolism, acting upstream of intramitochondrial ammonia production. Morin's inhibition of both basal and state IV respiration with malate‐glutamate as substrates points to a potential inhibition of glutamate dehydrogenase.

The role of glucose 6‐phosphatase inhibition by morin in reducing glucose output under glycogenolytic or gluconeogenic conditions is complex. As a terminal enzyme, glucose 6‐phosphatase exerts limited control over glucose output, shown by its low flux control coefficient. Its K_M_ for glucose 6‐phosphate is 3.5 mM, about 10 times higher than typical substrate levels [51, 89, 90], making its activity respond nearly linearly to increases in substrate concentration—such as those seen with morin. This rise tends to compensate for the enzyme's partial inhibition. Thus, glucose 6‐phosphatase inhibition has only a minor effect on total glucose output, with secondary importance. Supporting this, dihydroxyacetone‐driven gluconeogenesis showed only mild inhibition with morin, while glycogenolysis was actually stimulated at all tested concentrations.

In the presence of morin, deviations from the main glucose output pathway are likely, especially involving glucose 6‐phosphate and malate. Morin biotransformation includes glucuronidation, sulfation, and methylation via UDP‐glucuronosyltransferases, sulfotransferases, and catechol‐*O*‐methyltransferases [19]. This study also provides strong evidence that morin undergoes biotransformation in the microsomal electron transport system, which uses NADPH as an electron donor. In the cytosol, NADPH is mainly produced by malic enzyme and glucose 6‐phosphate dehydrogenase [61, 91], the latter inhibited by morin in vitro. The cyanide‐insensitive rise in oxygen consumption suggests this microsomal system remained active during morin treatment. The diversion of malate for NADPH production may partially account for the observed inhibition of the citric acid cycle under gluconeogenic conditions. Although this mechanism appears to play a secondary role in morin's action, it is a primary mode of inhibition for other compounds, such as aminopyrine [61, 91]. Glucuronide formation may also divert glucose 6‐phosphate, reducing glycolysis and glucose output. In fed rats, morin infusion increased biotransformation, indirectly confirming glucuronide formation [52]. When glucose 6‐phosphate dehydrogenase is inhibited, elevated glucose 6‐phosphate may enhance xenobiotic glucuronidation [92], potentially impairing glucose release, glycolysis, and gluconeogenesis as seen in this study.

Given morin's potential in treating metabolic complications associated with diabetes and obesity, such as hyperglycemia [17, 18, 19, 20], it is essential to assess whether the results observed in this study might impact in vivo physiological conditions. A key factor in addressing this question is determining the concentration of morin in the portal vein after oral administration, though such data is currently lacking. However, systemic levels of morin in rats have been documented. In one study, oral administration of a morin at 200 mg/kg resulted in a peak plasma concentration of approximately 3 μg/mL (around 10 μM) in rats [93]. When morin was orally administered as a morin‐phospholipid complex via a self‐nanoemulsifying drug delivery system to enhance absorption, plasma concentrations increased to approximately 25 μg/mL (around 82 μM) [93]. In the current study, the concentration of morin required for half‐maximal inhibition of gluconeogenesis from lactate and pyruvate was 162.66 μM. Given these considerations, it can be speculated that the observed effects are most relevant to toxicology and potential overdosing. However, it is worth that for many compounds, peak portal vein concentrations significantly exceed systemic levels, often by a factor of five or more [61, 94]. Additionally, morin appears to interact effectively with hepatocyte structures, as seen in the kinetics of effect reversal and elimination after infusion is stopped. The presence of a lag phase in both cases suggests gradual clearance from the cellular environment, implying that morin may accumulate in hepatic tissue. Although it is challenging to directly correlate dosages with plasma and tissue concentrations [69], it is plausible that similar effects could occur in vivo, particularly under pharmacological dosing of morin, such as through dietary supplements. In the fasted state, pharmacological administration of morin could be particularly beneficial for managing hyperglycemia in obese and diabetic patients, as glycemic control in this state depends heavily on hepatic gluconeogenesis [27, 28, 29, 30], which morin has been shown to acutely inhibit. It is unrealistic to expect only beneficial effects from morin, as its impact on mitochondrial bioenergetics in the liver is a significant part of its mechanism of action. Our study suggests that morin may effectively modulate glucose metabolism in the liver at lower concentrations and with short‐term exposure. However, at higher concentrations and prolonged exposure, morin's metabolic effects were linked to toxicity, evidenced by changes in the ATP/AMP ratio in the perfused liver, enzyme leakage, and decreased cellular viability in VERO and HepG2 cells. Our findings are consistent with previously reported cytotoxicity of morin, which is associated with its apoptotic potential [34, 35, 36]. Many of the adverse effects observed in this study are likely replicable in vivo, particularly with high doses and extended exposure periods. If such conditions occur, in addition to cellular damage, it is likely that glucose homeostasis will be disrupted due to inadequate suppression of gluconeogenesis and enhanced glycogenolysis, potentially altering amino acid metabolism. Moreover, it is crucial to recognize that the effects may not be limited to the liver, as mitochondrial metabolism is vital for ATP production in most cell types [95].

## 5 Conclusion

This study provides the first evidence of morin's acute modulation of glucose metabolism in the intact liver. The increase in glucose release via glycogenolysis, alongside suppressed gluconeogenesis, contrasts with the expected actions of antihyperglycemic drugs. This suggests that morin's stimulation of glycogenolysis may counteract its potential antihyperglycemic effects in vivo, possibly limiting its therapeutic value. Our results indicate that morin functions as a metabolic modulator with generally mild effects on liver metabolism. However, prolonged exposure or very high doses could pose serious risks to liver function and overall health. Therefore, in vivo studies using higher morin doses are essential to determine whether these effects persist, especially in the presence of hormones and other regulatory factors. Additionally, although our findings indicate a selective action of morin on specific key enzymes, as evidenced by the absence of effect on fructokinase in contrast to the significant inhibition of pyruvate kinase, glucose 6‐phosphate dehydrogenase, glucose 6‐phosphatase, and glucokinase, we cannot exclude the possibility of nonspecific interactions potentially leading to conformational changes in target proteins. Future studies evaluating direct interactions of morin with purified proteins using methods such as isothermal titration calorimetry, thermal shift assays, or circular dichroism could provide important additional insights into the specificity and structural mechanisms underlying the actions of this molecule.

## Conflicts of Interest

The authors declare no conflicts of interest.

## Data Availability

The data that support the findings of this study are available from the corresponding author upon reasonable request.
